# Reconciling nutritional geometry with classical dietary restriction: Effects of nutrient intake, not calories, on survival and reproduction

**DOI:** 10.1111/acel.12868

**Published:** 2018-11-20

**Authors:** Joshua P. Moatt, Murray A. Fyfe, Elizabeth Heap, Luke J. M. Mitchell, Fiona Moon, Craig A. Walling

**Affiliations:** ^1^ Institute of Evolutionary Biology, School of Biological Sciences University of Edinburgh Edinburgh UK; ^2^ Edinburgh Genomics, Roslin Institute University of Edinburgh Edinburgh UK

**Keywords:** caloric restriction, dietary restriction, life history, nutritional geometry, trade‐off

## Abstract

Dietary restriction (DR) is one of the main experimental paradigms to investigate the mechanisms that determine lifespan and aging. Yet, the exact nutritional parameters responsible for DR remain unclear. Recently, the advent of the geometric framework of nutrition (GF) has refocussed interest from calories to dietary macronutrients. However, GF experiments focus on invertebrates, with the importance of macronutrients in vertebrates still widely debated. This has led to the suggestion of a fundamental difference in the mode of action of DR between vertebrates and invertebrates, questioning the suggestion of an evolutionarily conserved mechanism. The use of dietary dilution rather than restriction in GF studies makes comparison with traditional DR studies difficult. Here, using a novel nonmodel vertebrate system (the stickleback fish, *Gasterosteus aculeatus*), we test the effect of macronutrient versus calorie intake on key fitness‐related traits, both using the GF and avoiding dietary dilution. We find that the intake of macronutrients rather than calories determines both mortality risk and reproduction. Male mortality risk was lowest on intermediate lipid intakes, and female risk was generally reduced by low protein intakes. The effect of macronutrient intake on reproduction was similar between the sexes, with high protein intakes maximizing reproduction. Our results provide, to our knowledge, the first evidence that macronutrient, not caloric, intake predicts changes in mortality and reproduction in the absence of dietary dilution. This supports the suggestion of evolutionary conservation in the effect of diet on lifespan, but via variation in macronutrient intake rather than calories.

## 1 INTRODUCTION

Understanding how diet influences traits such as aging, survival and reproduction is a fundamental question in biology with clear application to human health (Fontana & Partridge, 2015). Dietary restriction (DR), a reduction in the intake of calories or specific macronutrients whilst avoiding malnutrition, is the most consistent environmental manipulation to extend lifespan and delay aging (see Speakman & Mitchell, 2011; Selman, 2014 for recent reviews). However, the exact nutritional parameters responsible for the effect of DR are still unclear. In particular, there is considerable debate around the relative importance of calories versus macronutrient intake (see Speakman, Mitchell, & Mazidi, 2016; Ingram & de Cabo, 2017; Simpson et al., 2017). Recent work attempting to distinguish the effect of calories and macronutrient intake has been facilitated by the application of the geometric framework (GF) of nutrition, a state‐space based nutritional modelling method (Simpson & Raubenheimer, 2012; Simpson et al., 2017). The GF treats the diet as an *n*‐dimensional nutrient space, where n is the number of nutritional parameters. Any trait of interest can be plotted in this space to visualize the effect of multiple dietary components. By using a large number of diets varying in macronutrient and energy content, the effect of calories and specific macronutrients can be separated. A general pattern is emerging in insect literature, where macronutrient intake has a more prominent role than calorie content in determining survival, reproduction and the trade‐off between the two (e.g., Lee et al., 2008; Maklakov et al., 2008; Fanson, Weldon, Pérez‐Staples, Simpson, & Taylor, 2009; Jensen, McClure, Priest, & Hunt, 2015). Furthermore, in a rare application of the GF to a vertebrate species, it was the intake of protein and carbohydrate that determined lifespan in mice rather than overall calorie intake (Solon‐Biet et al., 2014), suggesting that the same patterns are true in vertebrates as well as invertebrates.

However, the importance of macronutrient intake in vertebrates is controversial (discussed Speakman et al., 2016; Ingram & de Cabo, 2017). The effect of protein intake in rodents is well studied, but often provides inconsistent results (reviewed Speakman et al., 2016; Ingram & de Cabo, 2017; Simpson et al., 2017). A comprehensive series of studies varying dietary protein content, but not using the GF, found that protein restriction could not produce the same effects as caloric restriction (e.g., Mitchell, Delville, et al., 2015; Mitchell, Tang, et al., 2015). The disparity between these studies and those of Solon‐Biet et al (2014) has been suggested to result from key methodological differences (Speakman et al., 2016). Studies utilizing the GF alter caloric intake through dietary dilution, reducing the energy content of diets, rather than restriction, reducing the amount of diet available (see Speakman et al., 2016). This has led to the suggestion of fundamental differences in the mode of action of DR between vertebrates and invertebrates, with more classical caloric restriction having a stronger effect in vertebrates as opposed to macronutrient content underpinning responses in invertebrates (Speakman et al., 2016). However, a meta‐analysis (Nakagawa, Lagisz, Hector, & Spencer, 2012) suggested the effect of protein on lifespan may be more consistent than the effect of calories, although this was based on data from experiments focussing on calorie restriction and so information on macronutrient intake was somewhat limited.

The suggestion of fundamental differences in the mode of action of DR between vertebrates and invertebrates questions the idea of an evolutionarily conserved mechanism and thus the use of DR as an experimental paradigm to understand the mechanisms underpinning lifespan and aging. However, vertebrate studies finding a stronger effect of calories tend not to use the GF and thus use fewer diets (e.g., Mitchell, Tang, et al., 2015), reducing the ability to distinguish the effect of calories from macronutrients (Simpson, Couteur, & Raubenheimer, 2015). Furthermore, the majority of studies comparing caloric restriction to macronutrient content in vertebrates have used laboratory strains of mice (e.g., Solon‐Biet et al., 2014; Mitchell, Tang, et al., 2015). The effect of DR has recently been shown to be stronger in laboratory model species than in nonmodel species (Moatt, Nakagawa, Lagisz, & Walling, 2016; Nakagawa et al., 2012), making general conclusions difficult to draw. Here using a novel nonmodel vertebrate system (the three‐spine stickleback (*Gasterosteus aculeatus*)), we provide, to our knowledge, the first test of the effect of macronutrient versus calorie intake on key fitness‐related traits that both uses the GF and avoids the potentially confounding effect of dietary dilution.

The effect of DR on lifespan is traditionally thought to be mediated by the trade‐off with reproduction as a result of direct competition for limiting resources between the two processes (Holliday, 1989; Shanley & Kirkwood, 2000). However, some recent results have challenged this assumption, with lifespan extension being observed either without an apparent reduction in reproduction or despite reproduction being physically, chemically or genetically prevented (e.g., Tu & Tatar, 2003; Mair, Sgro, Johnson, Chapman, & Partridge, 2004; Crawford, Libina, & Kenyon, 2007). Furthermore, it has been suggested that early life fitness traits can be enhanced without any significant lifespan cost, through use of exome‐matched diets (Piper et al., 2017). Studies using the GF suggest that rather than directly competing for limiting resources, lifespan and reproduction are instead maximized at different macronutrient intakes, resulting in a diet‐mediated trade‐off between the two (Jensen et al., 2015; Solon‐Biet et al., 2015). Again, the majority of these results come from studies of insects (e.g., Jensen et al., 2015, but see Solon‐Biet et al., 2015) and even here some studies suggest that lifespan and reproduction are maximized in remarkably similar areas of nutrient space (e.g., Maklakov et al., 2008). Further studies utilizing the GF and measuring both survival and reproduction, particularly in vertebrates, would be useful in determining the generality of trait specific macronutrient optima for survival and reproduction.

Traditional approaches to studying DR (manipulating calorie content) have suggested sex differences, with the effect of DR being stronger in females than in males (Burger & Promislow, 2004; Cooper, Mockett, Sohal, Sohal, & Orr, 2004; Magwere, Chapman, & Partridge, 2004; Nakagawa et al., 2012). This is suggested to result from females investing more in reproduction than males, but may instead be a result of males being exposed to less of the costs of reproduction than females in many experiments (see Moatt et al., 2016). In addition, recent studies using the GF suggest similarity between the sexes in the effect of diet on lifespan, but differences in the effect of diet on reproduction and the trade‐off between the two (Jensen et al., 2015; Maklakov et al., 2008). Direct comparisons of the sexes in the same study are rare, and rarer still are studies that use the GF to manipulate multiple aspects of the diet and expose both sexes to a range of reproductive costs.

Here, we address these issues by applying the GF to a wild‐derived population of three‐spined sticklebacks. Specifically, we address the following questions: (a) Is calorie or macronutrient intake the key determinant of mortality risk in a nonmodel vertebrate species? (b) Are survival and reproduction maximized at different macronutrient intakes suggesting a diet‐mediated trade‐off? and (c) Are there sex differences in the effect of macronutrient intake and calories on survival and reproduction when males experience more reproductive costs? We also explore other key fitness and health‐related traits, such as growth and body condition (e.g., Solon‐Biet et al., 2014; Moatt et al., 2017). Importantly, we manipulate calories by restricting diet availability (i.e., restriction) rather than via dilution (see methods). Overall we find support for the importance of macronutrient intake over calories in determining both mortality and reproduction. The effect of macronutrient intake on mortality was also sex‐specific (Table 1). Male mortality risk was lowest on intermediate lipid intakes, whilst female mortality risk was generally reduced by low protein intakes. However, the effect of protein on female mortality risk changed across ontogeny, being beneficial in early life and detrimental in late life. In both sexes, high protein intakes increased reproductive effort, providing evidence for a macronutrient mediated trade‐off between reproduction and mortality in sticklebacks.

**Table 1:** Summary of main results

Mortality risk		Reproduction		Reproductive senescence		Length		Condition	
Male									
Time Period (TP)	(+)	Protein	(+)	Age	(–)	Time Period (TP)	(+)	Time Period (TP)	(+/–)
Lipid	(–)			Age^2^	(–)	Protein	(–)	Lipid	(+)
Lipid^2^	(+)			Protein	(+)	Protein^2^	(–)	TP*Lipid	(+)
						Lipid	(+)	TP*Lipid^2^	(–)
						Lipid^2^	(–)	TP*Protein^2^	(–)
						TP*Lipid	(+)		
						Protein*Lipid	(+)		
Female									
TP	(+)	Protein	(+)	Age	(–)	TP	(+)	TP	(+/–)
TP*Protein	(+/–)	Lipid^2^	(–)	Age^2^	(–)	Lipid	(+)	Protein	(+)
				Lipid^2^	(–)	Lipid^2^	(–)	Lipid	(+)
				Age*Protein	(+)	Protein*Lipid	(+)		
				Protein*Lipid	(+)	TP*Lipid	(+)		
Sex‐Specific									
TP	(+ F)	NS		Age	(– M)	TP	(+ M)	TP	(+ M)
Lipid	(– M)			Age^2^	(– F)			Protein	(+ F)
Lipid^2^	(– M)							Lipid	(+ M)

Note

Only parameters with a significant effect are reported in this summary (*p < *0.05). Separate sex models were run to produce the **Male** and **Female** specific estimates, and then, the sex specificity of any particular effect was tested in a model that combined data from both sexes (**Sex‐Specific** above, see methods for details). + indicates a positive effect, – a negative effect, +/– represents effects that change over time (NB. for mortality risk – is a reduction in risk, and + is an increase in risk). For sex‐specific effects, the letter represents the sex where that specific effect was stronger (*M* = males, *F* = females) in the direction indicated by the symbol (+ (positive) or – (negative)). NS indicates none of the effects were significantly different between the sexes.^2^ indicates a nonlinear (quadratic) effect.

## 2 RESULTS AND DISCUSSION

We fed 300 male and 300 female individually housed three‐spine sticklebacks one of five diets varying in protein and lipid content (Table 2) at one of three provisioning levels (100%, 75% or 50% of ad lib), therefore using a restriction of food availability rather than a dilution of the diets to achieve calorie restriction. This gave a total of 15 dietary treatments (see methods and supplementary materials for full details). Fish were maintained on diets for life and measured for numerous traits including survival, reproductive investment, growth and body condition. Given the broad range of traits examined, we present data for each trait separately, with an accompanying short interpretation section. Broader patterns and implications of our results are discussed in the conclusion section.

**Table 2:** Nutrient content of the five diets used in this experiment

Protein (%)	Lipid (%)	Ratio P:L
67.5	6.6	10.2:1
33.2	3.9	8.5:1
59.3	13.0	4.6:1
51.6	20.5	2.5:1
31.2	19.2	1.6:1

### 2.1 Survival

Previous experiments have analysed lifespan against intake rates once growth has ceased and thus intake rates have stabilized (e.g., Lee et al., 2008; Solon‐Biet et al., 2014). This is not appropriate here as sticklebacks have indeterminate growth and thus intake rates vary over time. Therefore, as with a number of previous DR studies, we explore mortality risk (survival) rather than lifespan (e.g., Mair, Goymer, Pletcher, & Partridge, 2003; Colman et al., 2014). We analysed the effect of diet on survival using an event history analysis, which allows for time‐varying covariates and models how mortality risk varies over time and how this is affected by macronutrient intake and any other factors included in the model (see methods). This analysis is similar to that of a Cox proportional hazards model, but allows the more complex addition of time‐varying covariates. It provides us with a per time interval probability of death on the logit scale, which we term mortality risk throughout. Visual inspection of the mortality data showed clear variation in mortality risk across time (Supporting Information Figure S1). Therefore, the experiment was subdivided into 6 distinct periods where mortality risk noticeably varied (Supporting Information Figure S1).

#### 2.1.1 Findings

**Figure 1:**
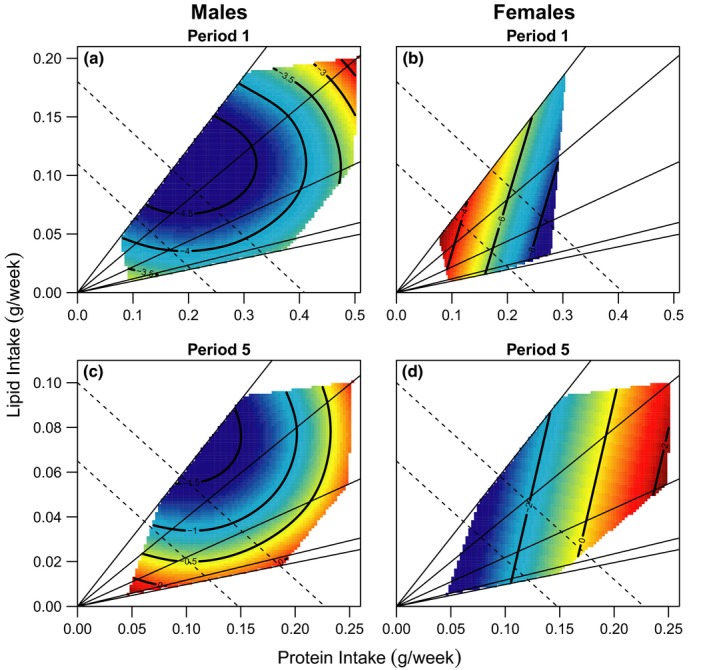
The effect of protein and lipid intake (g/week) on mortality risk in: males (a) and females (b) in time period 1, and males (c) and females (d) in time period 5. Positive values suggest high mortality risk; negative values suggest low risk. Mortality risks are given on the contours of each plot, with colours indicating an increasing risk from low risk (blue) to high risk (red). The five solid lines originating from the origin represent the five diets used in this experiment, and the dashed lines represent isocaloric intakes. There was a significant nonlinear effect of lipid on male mortality risk across all time periods. In females, there was an effect of protein on mortality risk; however, this changed over time, for example, being beneficial in time period 1 (b) and detrimental in period 5 (d), and these periods represent the extremes in the effect of protein. For further details on the interpretation of all contour plots, see Dialog S2

Male mortality risk varied over time (Supporting Information Figure S1) and was significantly affected by macronutrient intake. Male mortality risk was lowest on intermediate lipid intakes and increased as lipid intakes deviated from this point (Figure 1a,c, Supporting Information Table S1). This was consistent across all time periods (Figure 1a,c, Supporting Information Table S1). There was no effect of protein intake on male mortality risk (Figure 1a,c, Supporting Information Table S1). The effect of macronutrient intake was more important than calorie restriction in determining survival, with many diets showing no change in, or even increasing, male mortality risk with decreasing calorie intake (Figure 1a,c). This result is supported by additional analyses that demonstrated an effect of diet (i.e., macronutrient content), but not provisioning level (i.e., calorie restriction) on male mortality risk (Supporting Information Figure S2, Supporting Information Table S2).

Female mortality risk also changed over time (Supporting Information Figure S1) and was significantly affected by macronutrient intake. Although there was no overall effect of protein intake, there was a significant interaction between time period and protein intake on female mortality risk (Supporting Information Table S3). Increasing protein intake reduced female mortality risk in period 1 (Figure 1b, Supporting Information Table S3), prior to sexual maturity (weeks 0–9); but increased female mortality risk by period 5 (Figure 1d, Supporting Information Table S3), following cessation of reproductive activity (weeks 79–93). These two time periods represent the more extreme effects of protein on female mortality (Supporting Information Table S3). There was no effect of lipid intake on female mortality risk (Figure 1b,d, Supporting Information Table S3). The effect of macronutrient intake on female mortality risk appeared to be stronger than the effect of caloric intake (Figure 1b,d: Supporting Information Figure S2, Supporting Information Table S2). Although in time period 5 (Figure 1d) it appears that reducing intake reduces female mortality risk, it is clear that high mortality risk is confined to diets with high protein intakes.

Statistical comparison between the sexes demonstrated that the effect of macronutrient intake on mortality risk was sex‐specific (Table 3A). The beneficial effect of lipid was stronger in males than females, with a significant sex by lipid interaction (Table 3A). However, there was no evidence of a sex‐specific effect of protein intake on mortality (Table 3A), despite the suggestion of an effect of protein on female but not male mortality. Power issues prevented the fitting of a three‐way interaction between time period, sex and protein intake, so it is possible that there are sex differences in the effect of protein, but only in certain time periods. Males appear to live longer than females (Table 3A, Supporting Information Figure S1), but this may be a result of males not being exposed to direct physical competition with other males during the breeding season.

**Table 3:** The sex‐specific effect of macronutrients on mortality (A) and reproduction (B)

	Estimate (± *SE*)	*χ* ^2^	*p*
(A) Mortality risk			
*Intercept*	−5.165 (0.293)		
Time Period 2	0.087 (0.315)		
Time Period 3	1.879 (0.343)		
Time Period 4	1.442 (0.380)		
Time Period 5	3.423 (0.466)		
Time Period 6	3.815 (0.629)		
Protein	0.021 (0.100)		
Lipid	−0.073 (0.241)		
Lipid^2^	0.067 (0.218)		
Sex (male)	0.766 (0.295)		
Protein*Sex	0.138 (0.156)	0.79	0.373
Lipid*Sex	−1.02 (0.379)	3.83	0.050
Lipid^2^*Sex	0.754 (0.353)	4.66	0.031
Time Period 2*Sex	−1.198 (0.383)		
Time Period 3*Sex	−1.419 (0.365)		
Time Period 4*Sex	−1.945 (0.368)		
Time Period 5*Sex	−1.867 (0.37)		
Time Period 6*Sex	−2.573 (0.516)	43.59	<0.001
(B) Reproduction			
*Intercept*	0.014 (0.082)		
Protein	−0.093 (0.304)		
Lipid	0.543 (0.235)		
Protein^2^	0.189 (0.309)		
Lipid^2^	−0.492 (0.235)		
Sex (male)	−0.033 (0.088)		
Protein*Sex	0.462 (0.410)	1.04	0.307
Protein^2^*Sex	−0.370 (0.412)	0.82	0.366
Lipid*Sex	−0.134 (0.356)	1.39	0.239
Lipid^2^*Sex	0.010 (0.357)	0.00	0.973

Note

Mortality outputs are from an event history model (binomial GLME); model contains main effects that were significant in split sex models (see Supporting Information Tables S1 and S2) and their interactions. Reproduction outputs from LME model. Female reproduction = total egg production, male reproduction = total courtship (s).

#### 2.1.2 Implications

The importance of macronutrient intake over calories in determining male mortality supports previous findings in insects (Jensen et al., 2015; Maklakov et al., 2008) and one in mice (Solon‐Biet et al., 2014), showing significant nonlinear effects of nonprotein dietary components on male survival, and that survival is maximized on low protein content diets. Interestingly, these diets increase adiposity (Solon‐Biet et al., 2014), and it has been demonstrated that fat deposition increases with increasing dietary lipid content in sticklebacks (Moatt et al., 2017). These results either provide support to previous challenges of a link between a reduction in adiposity and an increase in lifespan under DR (Barzilai, Banerjee, Hawkins, Chen, & Rossetti, 1998; Muzumdar et al., 2008; Picard & Guarente, 2005) or suggest that low protein diets have a beneficial effect on lifespan despite causing an increase in adiposity and its associated negative effects on health (Le Couteur et al., 2016).

Higher mortality in females with higher protein intakes, rather than at higher caloric intake, also supports recent literature (e.g., Lee et al., 2008; Maklakov et al., 2008; Fanson et al., 2009; Solon‐Biet et al., 2014; Jensen et al., 2015). However, the effect of protein intake on early life survival contrasts with these previous results. One explanation for this difference is in how our data were analysed. In previous studies, intakes were quantified over a time period where growth had ceased and intakes were stable (e.g., Solon‐Biet et al., 2014). This period typically corresponds to an adolescent/adult period, where growth has stopped, rather than juvenile or early life, where growth rates are high. Therefore, it is possible that a beneficial early life effect of protein intake has been overlooked in previous studies that generally ignore early life, where the diet that optimizes survival may be different. In line with this hypothesis, in *Drosophila melanogaster*, it has been suggested that egg to pupae survival is maximized on high protein content diets (Rodrigues et al., 2015 but see Davies et al., 2018), in contrast to adult lifespan, which was maximized on low protein content diets (Lee et al., 2008). Previous research into DR has focussed on later life survival and aging; from the work that does exist, the effect of early life diet on lifespan appears to be small, but may be stronger in vertebrates than invertebrates (English & Uller, 2016). By applying survival analyses that allow time‐varying covariates, we were able to detect an early life benefit of protein to immediate mortality risk. It would be interesting to apply these analytical techniques in other species to test whether the effect of protein changes across ontogeny.

In general, previous studies have reported that the effect of macronutrient intake on mortality risk is similar across the sexes (Solon‐Biet et al., 2014; Jensen et al., 2015, but see Maklakov et al., 2008). However, studies involving direct comparisons are rare. Even the sex differences reported by Maklakov et al. (2008) were driven by slight sex differences at very high carbohydrate intakes. In our study, there were more fundamental differences between the sexes, with male mortality being strongly affected by lipid intake whilst female mortality was affected by protein intake—although this effect was variable across time. Explanations for sex differences in the effect of diet on survival centre on differences in the reproductive costs faced by males and females (Moatt et al., 2016), and this seems likely here. Typically, DR experiments do not expose males to a full range of reproductive costs, such as repeated courtship attempts and intrasex competition (e.g., male *D. melanogaster* in Jensen et al., 2015), whereas females generally are exposed to egg laying, which is presumably a major cost of reproduction (see Moatt et al., 2016). It has been suggested that this impacts on our ability to detect shifts in male mortality (Moatt et al., 2016). We suggest that, by exposing both females and particularly males to more of the costs of reproduction (e.g., courtship, territory defence, nuptial coloration and nest building) than other studies, our study has accentuated the differences in the effect of macronutrient intake on mortality risk between the sexes (Moatt et al., 2016). However, more studies comparing the effect of diet on mortality risk between the sexes, particularly where both sexes are exposed to near complete reproductive costs, are needed to test this hypothesis.

### 2.2 Lifetime reproductive investment

#### 2.2.1 Findings

**Figure 2:**
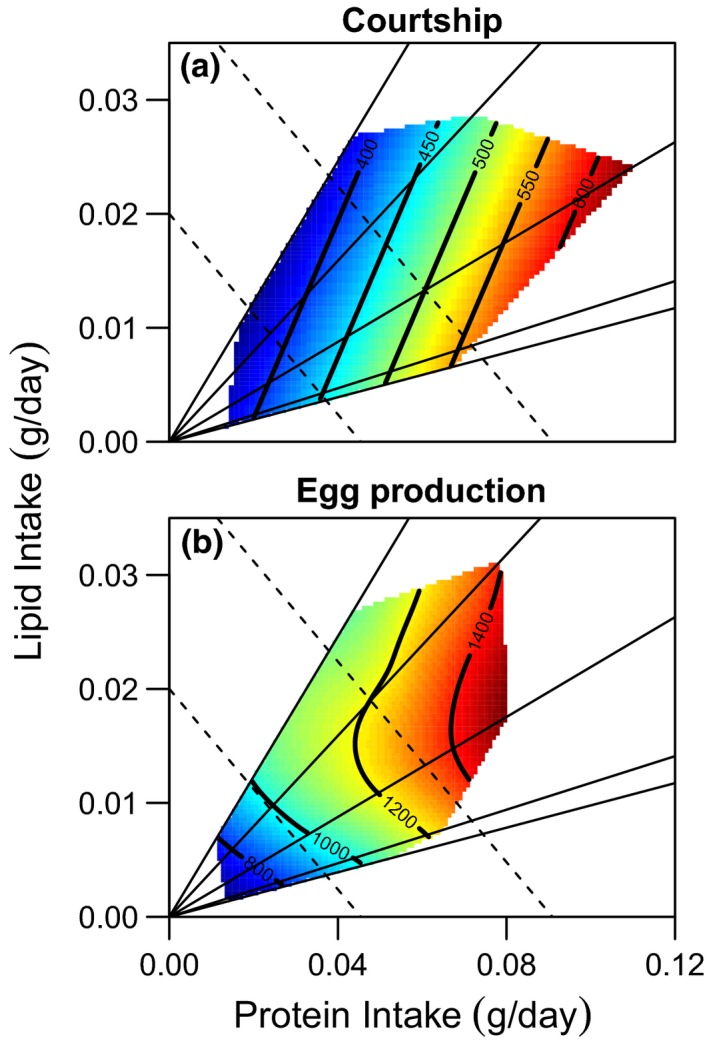
The effect of protein and lipid intake on: (a) Courtship (time courting (s)) and (b) Egg Production (total number of eggs laid). Time spent courting and number of eggs are given on the contours of each plot, respectively, with colours indicating increasing investment in reproduction from low (blue) to high (red). The five solid lines originating from the origin represent the five diets used in this experiment, and the dashed lines represent isocaloric intakes. The effect of macronutrient intake on reproduction was not sex‐specific, with both males and females maximizing reproduction at higher protein intakes

Male investment in reproductive behaviour (time spent courting) was significantly greater on high protein intakes (Figure 2a, Supporting Information Table S4). There was no detectable effect of lipid intake on time spent courting, although the nonlinear effect of lipid intake was marginally nonsignificant (Figure 2a, Supporting Information Table S4). The same general patterns were observed for other measures of courtship investment (Supporting Information Table S5) and measures of territory defence (Supporting Information Table S6). In contrast, there was no suggestion of an effect of macronutrient intake on the number of nests attempted or completed (Supporting Information Table S7).

Female reproduction (total egg production) was maximized at high protein intakes (Figure 2b, Supporting Information Table S8). However, there was also a nonlinear effect of lipid intake, with egg production highest at intermediate lipid intakes (Figure 2b, Supporting Information Table S8). This increase in total egg production was due to an increase in both the size and number of clutches produced by females on high protein and intermediate lipid intakes (Supporting Information Table S9).

Despite intermediate lipid intakes increasing egg production in females and no apparent effect of lipid on courtship in males, we found no evidence for sex‐specific effects of macronutrient intake on reproductive investment (Figure 2, Table 3B). This contrasts the sex‐specific effect of macronutrient intake on mortality risk detailed above.

#### 2.2.2 Implications

In females, reproduction is generally maximized on high protein intakes (e.g., Lee et al., 2008; Maklakov et al., 2008; Jensen et al., 2015) and our results support this. However, previous results in males are inconsistent, with some finding male reproductive investment is greater on diets with high protein contents (e.g., Hunt et al., 2004; Solon‐Biet et al., 2015), whilst others find lower protein intakes benefit male reproduction (Jensen et al., 2015; Maklakov et al., 2008). One possible explanation for this difference is the type of reproductive trait measured, with energetically expensive traits perhaps requiring lower protein diets (Maklakov et al., 2008 but see Hunt et al., 2004). Interestingly, we would expect courtship, our measure of reproductive investment, to be energetically expensive. One explanation for the lack of a lipid (nonprotein energy) effect of diet in our study is linked to breeding behaviour in the wild. Male sticklebacks are unlikely to forage during the breeding season in the wild (Rohwer, 1978) and thus may store lipid in advance (see Moatt et al., 2017). In our study, males were not food limited during the breeding season, and it is therefore possible that males were able to utilize lipid stores for reproduction. Thus, it is possible no males, even those on the lowest lipid diets, were actually limited in lipid availability during the breeding season. Instead, males on high protein diets invested more in courtship, perhaps because protein improved some other determinant of reproduction such as sperm quality or hormone levels (Solon‐Biet et al., 2015), stimulating males to court more.

Coupled with the effect of diet on mortality risk, our results suggest that diet may mediate the trade‐off between reproduction and survival in both sexes. Reproduction appears to be maximized at higher protein intakes in both sexes, whereas survival is maximized at intermediate lipid intakes in males and generally at low protein intakes in females. Thus, the dietary optima for survival and reproduction mismatch in both sexes. These results fit well with those generally reported in the literature, with reproduction often maximized at high protein intakes, survival maximized at low protein intakes and fitness maximized at an intermediate point (e.g., Hunt et al., 2004; Lee et al., 2008).

There was no suggestion of a sex‐specific effect of macronutrient intake on reproduction. This finding is consistent with the only previous result in a vertebrate (Solon‐Biet et al., 2015), but contrasts with invertebrate results where reproduction in females was maximized on diets with higher protein contents than males (Jensen et al., 2015; Maklakov et al., 2008). The explanation for the difference between studies is unclear, but all studies suggest the existence of an optimal intake of protein to nonprotein energy in the diet, with measures of reproduction decreasing on intakes above and below this (Jensen et al., 2015; Maklakov et al., 2008; Solon‐Biet et al., 2015).

### 2.3 Reproductive senescence

#### 2.3.1 Findings

**Figure 3:**
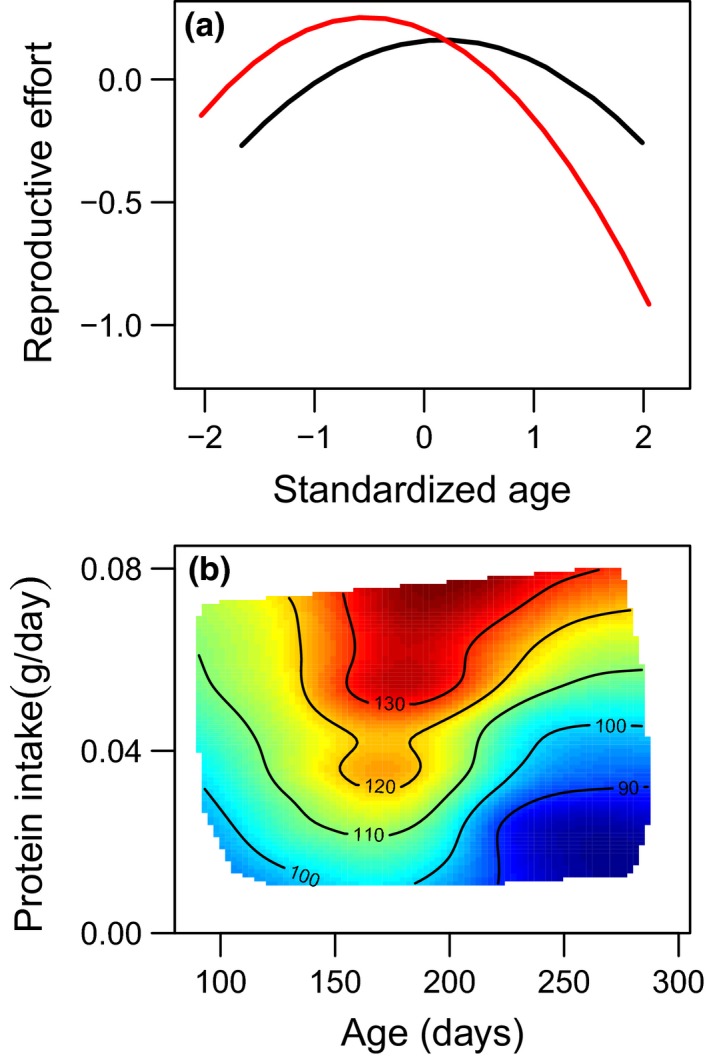
(a) Predicted sex‐specific trajectories of reproduction. Females (red) have a higher initial reproductive effort than males (black), however, suffer a much faster rate of decline with age. The age ranges used for these curves were chosen to cover the 90th percentile of the data. Age and reproductive effort are standardized values (mean of 0 and standard deviation of 1), to allow direct comparison between males and females. (b) The effect of protein on female egg production with age. Egg number is given on the contours of (b), with colours indicating an increase in egg production from low (blue) to high (red). Higher protein intakes resulted in slower declines in reproduction with age. As there was no effect of macronutrient intake on male reproductive senescence, we do not include a panel for males (see Supporting Information Figure S3 for male plots)

There was a significant nonlinear effect of age on courtship investment in males, with investment in courtship increasing initially but declining at older ages (Figure 3a, Supporting Information Table S10). There was no effect of either lipid or protein intake on male reproductive senescence (Supporting Information Figure S3, Supporting Information Table S10), despite a positive linear effect of protein on investment in courtship (see above). There was a negative effect of age of first reproductive event on investment, with males that started reproducing later in life having lower investment in courtship (Supporting Information Table S10).

There was also a nonlinear effect of age on female reproductive investment, with clutch size increasing to a peak at intermediate ages and then declining in old age (Figure 3a, Supporting Information Table S11). There was a significant effect of protein intake on female reproductive decline, (Figure 3b, Supporting Information Table S11), but no effect of lipid intake (Supporting Information Figure S3, Table S11). Individuals with higher protein intakes had a slower rate of reproductive senescence than those with lower protein intakes. There were additional effects of age of first and age of last reproductive attempt on clutch size as well as interactions between these and the nonlinear effect of age (see Supporting Information Figure S4 and supplementary results and discussion).

There were significant differences in the patterns of senescence between the sexes (Figure 3a, Supporting Information Table S12), with females having higher initial reproductive effort than males, but suffering a much faster rate of reproductive decline (Figure 3a). However, there was no difference in the effect of protein or lipid intake on reproductive senescence between the sexes (Supporting Information Table S12).

#### 2.3.2 Implications

The beneficial effect of protein intake on female reproductive senescence reported here is consistent with two recent studies in insects (Jensen et al., 2015; Maklakov et al., 2009), where reproductive senescence in females was also lower at the highest level of protein intake (although this effect was not significant in Maklakov et al., 2009). However, in contrast to these studies (Jensen et al., 2015; Maklakov et al., 2009), we found no evidence of a sex‐specific effect of macronutrient intake on senescence, although the effect of protein intake on senescence was only significant for females. This fits with our finding of a lack of sex differences in the effect of macronutrient intake on lifetime reproduction, whilst contrasting other studies reporting sex differences in the effect of macronutrient intake on both lifetime reproduction and reproductive senescence (Maklakov et al., 2009; Jensen et al., 2015, but see Solon‐Biet et al., 2015). The reason for these differences is unclear, but may be due to the different reproductive traits that have been measured in different studies (see above). Future studies should attempt to focus on measures of reproduction that are most relevant to the natural ecology of the study organism, although achieving such measures can often be practically very challenging.

### 2.4 Growth

#### 2.4.1 Findings

**Figure 4:**
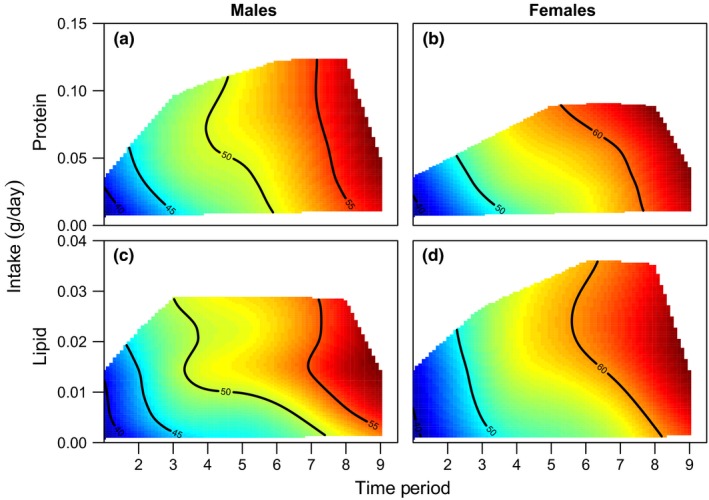
The effect of protein and lipid intake (g/day) on length (mm) across time periods. Effect of: (a) protein on male length, (b) protein on female length, (c) lipid on male length and (d) lipid on female length. Length is given on the contours of each plot, with colours indicating an increasing length from short (blue) to long (red). Both males and females maximized length on diets that contained relatively high levels of both protein and lipid. This effect of macronutrient intake changed over time in both sexes, for example, high protein intakes increased male length at time period 2, but intermediate protein intakes increased length in time period 5 (panel A). See Supporting Information Figure S5 for the effect of protein and lipid on length split by time period

Growth is likely to be positively correlated with reproduction in sticklebacks (larger males are better competitors, larger females produce more eggs) and may therefore also mediate the relationship between diet and lifespan (Wootton, 1973, 1984 ). We use change in fish length as our measure of growth. Male length increased over time (Figure 4; Supporting Information Figure S5, Table S13). For both protein and lipid, intermediate intakes resulted in greater increases in male length (Figure 4; Supporting Information Figure S5, Table S13). There was a significant interaction between lipid intake and time period (Figure 4; Supporting Information Figure S5, Table S13), with the positive effect of lipid intake on male length becoming stronger over time. Additionally, there was a significant interaction between protein intake and lipid intake (Supporting Information Table S13), suggesting the effect of lipid intake on male length was greater with higher protein intakes and vice versa (Supporting Information Figure S5). These patterns were similar for male weight (Supporting Information Figure S6, Table S14).

Female length also increased over time (Figure 4; Supporting Information Figure S5). As with males, intermediate lipid intakes resulted in greater increases in female length (Figure 4; Supporting Information Figure S5, Table S15). However, there was no detectable effect of protein intake on female length (Figure 4; Supporting Information Figure S5, Table S15). There was a significant interaction between lipid and time period (Supporting Information Table S15), suggesting the positive effect of lipid on female length increased in strength across time periods. Finally, there was a significant interaction between protein intake and lipid intake on female length (Supporting Information Table S15), suggesting the effect of lipid intake was greater with higher protein intakes and vice versa (Supporting Information Figure S5). There were some differences between the effects of macronutrient intake on female weight (Supporting Information Figure S6, Table S16) to those on female length described here, but the overall patterns remained the same.

There was no significant difference between the sexes in the effect of macronutrient intake on length (Table S17, Figure 4; Supporting Information Figure S5), and the same was true for weight (Supporting Information Figure S5, Table S18). However, there were significant differences in size between the sexes (see supplementary analysis and Supporting Information Figure S7).

#### 2.4.2 Implications

Previous work has focused on the relationship between reproduction and lifespan given the suggested shift in the lifespan—reproduction trade‐off under DR (Shanley & Kirkwood, 2000). However, growth is also well known to trade‐off with lifespan (Charnov, Turner, & Winemiller, 2001). Here, as often in studies of fish species, we use change in length as a measure of growth (e.g., Inness & Metcalfe, 2008). The results presented here mirror those of Solon‐Biet et al. (Solon‐Biet et al., 2014), suggesting a lack of diet‐mediated trade‐off between growth and survival. The diets producing the highest growth did not also result in the highest mortality. In males, intermediate lipid intakes improved both mortality risk and growth. Furthermore, in female early life, protein intake has a positive effect on growth and reduced mortality risk.

### 2.5 Body condition

#### 2.5.1 Findings

**Figure 5:**
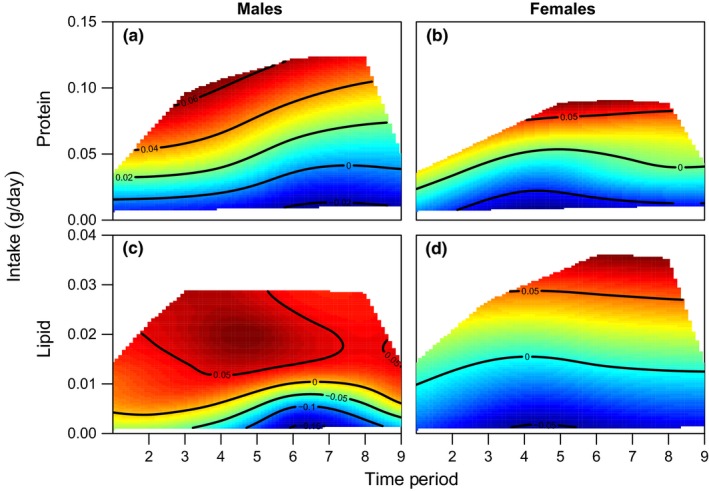
The effect of protein and lipid intake (g/day) on fish condition across time periods. Effect of: (a) protein on male condition, (b) protein on female condition, (c) lipid on male condition and (d) lipid on female condition. A positive value represents better than average condition; negative is worse than average. Condition scores are given on the contours of each plot, with colours indicating an increasing condition from low (blue) to high (red). Generally, male condition was improved on intermediate lipid intakes, though this effect changed over time. Female condition was greater on high protein and high lipid intakes which was consistent over time. Although the surfaces look similar between the sexes, the magnitude of the effect of macronutrients was significantly different (see Supporting Information Tables S19–S21): with lipid improving condition to a greater extent in males than females, and the opposite being true for protein intake. See Supporting Information Figure S8 for the effect of protein and lipid on body condition split by time period

As a proxy for overall health, we use body condition index, which is a measure of the weight of an individual relative to its length (see supplementary methods for full details). Here, a negative value indicates an individual weighing less than average for its length, whilst a positive value suggests an individual weighing more than average for its length. Male body condition varied over time (Figure 5; Supporting Information Figure S8, Table S19). High lipid intakes improved male condition (Figure 5; Supporting Information Figure S8, Table S19). However, this effect varied over time, with intermediate lipid intakes being more beneficial for male condition at some time points (Supporting Information Table S19). There was no overall effect of protein intake on male condition (Supporting Information Table S19); however, there was evidence of a beneficial effect of intermediate protein intakes at some time points (Figure 5; Supporting Information Figure S8, Table S19). Female condition also varied over time (Figure 5; Supporting Information Figure S8, Table S20) and was improved by high protein intakes and high lipid intakes (Figure 5; Supporting Information Figure S8, Table S20). There was no evidence that these effects changed over time (Figure 5; Supporting Information Figure S8, Table S20). There were sex‐specific effects of macronutrient intake. Lipid intake had a stronger effect on male condition than on female condition (Supporting Information Table S21). Conversely, protein intake had a stronger effect on female condition than on male condition (Supporting Information Table S21). There was also significant sexual dimorphism in condition (see Supporting Information Figure S7 and supplementary analysis).

#### 2.5.2 Implications

The effects of macronutrient intake on mortality and body condition and the results of a previous study on lipid deposition (Moatt et al., 2017) suggest a possible link between lipid intake, adiposity, health and survival in male sticklebacks. Intermediate lipid intakes result in higher adiposity and better overall health (as indicated by improved body condition above) and reduced risk of mortality. It would be interesting to see how other measures of health in sticklebacks are affected by intermediate lipid intakes and in particular, whether these intakes improve all measures of stickleback health. Furthermore, it would be interesting to see whether the diets resulting in the greatest lifespan, such as low protein content diets, also improve aspects of health in other species (e.g., Solon‐Biet et al., 2014) and whether low protein diets improve lifespan despite increasing adiposity or whether these levels of adiposity represent “healthy obesity” (Le Couteur et al., 2016).

### 2.6 Intakes

As well as affecting key life history traits, diet composition can also affect intake rates, which can have important feedback effects on health and lifespan (Fanson et al., 2009; Solon‐Biet et al., 2014). As a result of the restriction methods we used, only individuals on the 100% diet could choose their intake rate. Therefore, we cannot test the independent effect of protein and lipid content of the diet on intake as we only have natural intake rates for the five diets at 100%. Instead, we investigated the effect of diet as a factor on intake rate and interpret this based on the constituents of the diets (Fanson et al., 2009; Solon‐Biet et al., 2014, see methods for full details). There was a significant effect of both diet and size of the fish on intake rate, but no differences in intake rates between the sexes (Supporting Information Figure S9, Table S22). Fish consumed the most on diets with an intermediate protein and lipid content, with the highest intake being achieved on the diet containing 59% protein and 13% lipid (Supporting Information Figure S9, Table S23). As the protein and lipid contents deviated from this, there was a reduction in intake rate (Supporting Information Figure S9, Table S23).

## 3 CONCLUSIONS

It is widely accepted that DR, a reduction in the intake of food or particular nutrients whilst avoiding malnutrition, increases lifespan at the expense of reproduction, and that this effect is stronger in females than males (reviewed Speakman & Mitchell, 2011; Nakagawa et al., 2012; Selman, 2014). However, growing evidence suggests that macronutrient intake rather than restriction of caloric intake underpins this effect (reviewed Nakagawa et al., 2012; Simpson et al., 2017) and that under macronutrient manipulation sex differences are less pronounced (Jensen et al., 2015; Maklakov et al., 2008). The majority of this evidence comes from studies of insects, and the importance of dietary macronutrient intake has rarely been tested in vertebrates. In addition, those studies that do exist suffer from methodological differences that make general conclusions difficult to draw (Solon‐Biet et al., 2014; Mitchell, Tang, et al., 2015; Speakman et al., 2016 see introduction above). We present an empirical study that directly tests the effect of dietary macronutrient intake against calorie intake in a nonmodel vertebrate species and, critically, uses the GF and avoids the potentially confounding effect of dietary dilution (see Speakman et al., 2016). Overall, we found that mortality risk, reproduction, growth and health (body condition) are determined more by macronutrient intake, than calorie intake (Table 1). These results challenge the suggestion of fundamental differences in the mode of action of DR between vertebrate and invertebrate species (Speakman et al., 2016) and support previous suggestions of a benefit of low protein diets for survival and lifespan (Nakagawa et al., 2012; Simpson et al., 2017; Solon‐Biet et al., 2014). Our results also provide novel evidence of sex differences in the effect of macronutrient intake on mortality risk. We provide evidence of macronutrient mediated trade‐offs between survival and reproduction, with these traits maximized at different macronutrient intakes (Table 1). However, we do not find evidence of a trade‐off between growth and survival, with the macronutrient intakes that maximized growth not resulting in reduced survival. Thus, our experiment provides key support for the hypothesis that fitness‐related traits are more determined by macronutrient intake than calorie intake and that this effect may be consistent across vertebrates and invertebrates (Nakagawa et al., 2012). Such conservation is key if we are to use DR research to understand the mechanism determining variation in lifespan, reproduction and aging (Fontana & Partridge, 2015; Piper & Partridge, 2017; Selman, 2014).

## 4 EXPERIMENTAL PROCEDURES

A detailed description of the methods used can be found in the supplementary file.

### 4.1 Husbandry

A total of 600 (300 of each sex) first‐generation offspring of wild‐caught three‐spine sticklebacks were split equally across 15 dietary treatments (*n* = 20 of each sex per treatment, Supporting Information Table S24). The same diets were used as in Moatt et al. (2017) which varied in protein and lipid content (Table 1, Supporting Information Table S25). Carbohydrate (corn starch) was included in the diets as a filler to allow the independent variation of protein and lipid in the diets as this is indigestible to predatory fish such as sticklebacks (Moatt et al., 2017 and see supplementary methods). These five diets were provided at one of three levels: 100% (ad libitum), 75% and 50% of *ad lib*, giving a total of 15 dietary treatments. With the 100% treatment fed twice a day, the 75% treatment fed alternately once a day and then twice on the second day and the 50% treatment fed once a day, with feeding levels quantified through monthly monitoring of sentinel fish (see supplementary methods). This intermittent feeding regime resulted in a restriction in intake (i.e., a restriction of calories consumed), rather than a dilution of the diet (e.g., using diets varying in calorie density).

### 4.2 Data collection

Throughout the experiment, fish were monitored for a number of key traits including: mortality, reproduction, growth and condition. Mortality was checked twice daily and date of death was recorded. We quantified male lifetime reproductive investment as the total time spent courting (in seconds) across all courtship attempts. Males were also assayed for other common reproductive behaviours (e.g., territory defence, nesting and nuptial coloration—see supplementary methods and analysis). Female lifetime reproductive investment was taken as the total number of eggs produced). Fish were monitored for growth (length (mm) and weight (g)) approximately every 1–2 months (Supporting Information Table S26). From these measures, body condition (overall health) was also quantified (Moatt et al., 2017 and supplementary methods).

### 4.3 Statistical analysis

All analyses were carried out in R (v3.4.0, R core team, 2017) using the packages *fields*,* lme4* (Bates, Mächler, Bolker, & Walker, 2015) and *ASReml‐R* (v3.0; Gilmour, Gogel, Cullis, Thompson, & Butler, 2009). We used a response‐surface approach (Lande & Arnold, 1983) to estimate the linear and nonlinear (quadratic) effects of protein and lipid intake and the interaction between them (e.g., Jensen et al., 2015, see supplement). For all analyses, protein and lipid intakes were standardized to a mean of zero and a standard deviation of one to avoid issues of scale differences when fitting quadratic terms. Nutritional landscapes were visualized using thin‐plate splines. Full details of analyses and model specification are provided in the supplementary materials.

As detailed above, survival was analysed using an event history analysis through generalized linear mixed models (GLME). This is similar to a Cox proportional hazards model, but allows for the use of time‐varying covariates (see supplementary methods for full details). This analysis provides us with a per time interval probability of death on the logit scale, which we term mortality risk. There was clear variation in mortality risk across time (Supporting Information Figure S1), and therefore, we subdivided the experiment into six distinct periods where mortality varied. These time periods were based on visual inspection of mortality data and represent periods where mortality noticeably changed.

Measures of total reproductive investment were analysed using linear mixed effects models (LME). As intake rates were stable throughout the breeding season, we analysed the average daily intake (g/day) of protein and lipid across the course of the breeding season. We also used LME models to explore the effects of protein and lipid intake on age‐specific reproduction (reproductive senescence) in both sexes.

Weight, length and body condition were analysed through LME models using ASReml‐R (see supplementary methods). Protein and lipid intakes were calculated as the average daily intake (g/day) for the period between each measurement (i.e., the average daily intake between weighing 1 and weighing 2). All models included time period as a factor, with protein and lipid being interacted with time period to test for changing effects over time.

Intakes from a period of stable intake between days 263 and 458 of the experiment (stable across 195 days) were analysed using general linear models, in an adaptation of previous methods (Fanson et al., 2009). As only sentinel fish can select their own intake rates for the five diets (see supplementary methods), we have insufficient data to test the independent effect of protein and lipid content of the diet on self‐selected intake. Note, exact intake rates are available for all individuals, but individuals other than sentinel individuals were fed a specific ration determined by the sentinel individuals, and thus, this is not a self‐selected intake. Models exploring intake rates included diet as a categorical fixed effect.

## CONFLICT OF INTEREST

None declared.

## AUTHOR CONTRIBUTIONS

CAW and JPM conceived and designed the study. JPM led the data collection and all authors participated in data collection. Statistical analysis was carried out by JPM and CAW. JPM wrote the initial draft of the manuscript, with CAW and JPM performing revisions. All authors approved the final version of the manuscript.

## Data Availability

All data and scripts associated with this work are stored on the dryad repository (https://doi.org/10.5061/dryad.g12p0j2).
