# Supplementary material for: Reconciling nutritional geometry with classical dietary restriction: Effects of nutrient intake, not calories, on survival and reproduction
Source: Aging Cell. 2018 Nov 20;18(1):e12868. doi: 10.1111/acel.12868 (PMC6352320; doi:10.1111/acel.12868)
Supplement: Supplementary file 1 [file ACEL-18-e12868-s001.docx]

Reconciling nutritional geometry with classical dietary restriction: effects of nutrient intake, not calories, on survival and reproduction.

Joshua P. Moatt^1^*, Murray A. Fyfe^1^, Elizabeth Heap^2^, Luke J. M. Mitchell^1^, Fiona Moon^1^ & Craig A. Walling^1*^.

Dialog S1: Supplementary Methods

**Husbandry**

Experimental individuals were first generation offspring of wild caught three-spine sticklebacks. Parent fish were collected from Inverleith Pond, Edinburgh (55.96N 3.22W) during the spring of 2014. Using standard IVF techniques (Barber & Arnott 2000), 23 clutches were produced, each from a unique sire and dam. Offspring were maintained following the same procedure as that of Moatt *et al.* (2017) until 4 months of age, when fish were molecularly sexed through fin clips, weighed, and then assigned to one of 15 diet treatments. Initially, a total of 600 individuals, 300 of each sex, were split equally across diets (n= 20 per sex, per dietary treatment). However, due to mortality prior to the start of dietary manipulations, a total of 594 individuals were used in the experiment (see Table S24 for breakdown of sample sizes).

Fish were housed in flow through glass sided aquaria split into compartments (7 x 25 x 50cm) housing a single fish and containing an artificial plant. Compartments were created using opaque semi-permeable plastic divides, allowing water movement but preventing the movement of fish or food debris. Temperature and light regimes were matched to the natural levels for Edinburgh at that time of year. Clutches and treatments were evenly distributed between stacks and shelves to control for both family and tank effects.

**Dietary Treatments**

The composition of diets used in this experiment were the same as those used in Moatt *et al.* (2017, and see Tables 1 and S25). Geometric framework (GF) studies of rodents and insects typically focus on the protein and carbohydrate contents of diets (e.g. Lee *et al.* 2008; Maklakov *et al.* 2008). However, carbohydrate is not a key macronutrient for predatory fish such as sticklebacks (Ruohonen *et al.* 2003; Ruohonen *et al.* 2007; Moatt *et al.* 2017). We therefore chose to vary the protein and lipid content of the diet. Carbohydrate was present in our diets in the form of an inert filler (corn starch) that was indigestible to sticklebacks (Table S25). A total of five diets varying in protein and lipid content were used (Table 2). These macronutrient levels were selected through examination of the nutrient content of typical stickleback prey items, and were designed to sample the ranges experienced by sticklebacks naturally. The diets were specifically designed so that there was a lack of correlation between the energy density and the protein : lipid ratio of the diets. This was to facilitate analysis and allow us to disentangle the effects of macronutrient intake and energy content (e.g. Moatt *et al.* 2017). All diets were designed to have an excess of carotenoid so that individuals on the smallest ration would ingest sufficient carotenoid to display nuptial colour.

Each of the five diets were provided at one of three levels; 100% (*ad libitum*), 75% and 50% of *ad lib*, giving a total of 15 dietary treatments. To avoid problems associated with dietary dilution and the additional problem of rapid food degradation in water, we used an intermittent feeding regime which has previously been used to successfully implement DR in fish (e.g. Terzibasi *et al.* 2009; Moatt *et al.* 2017). Individuals in the 100% treatment were fed twice a day, the 75% treatment were fed alternately once a day and then twice on the second day and the 50% treatment were fed once a day. Thus the reduction in caloric intake here is generated through a reduced feeding quantity, rather than a dilution of the calorie density within the diet, facilitating the separation of the effect of macronutrients from calories. Feeding levels were quantified using monthly monitoring of sentinel fish for each diet from the 100% treatment (see below). Diets were initiated 24/11/2014 when fish were 172.24 ± 0.08 days old and individuals were maintained on diets for the rest of life.

To restrict the amount of food eaten as well as varying macronutrient content, individual fish were fed a specific ration across the experiment (Terzibasi *et al.* 2009). To account for differences in feeding rate between fish of different size, we classified fish of each treatment as either large (heaviest 10 fish) or small (lightest 10 fish) for each sex. Thus there were 60 different feeding quantities (sex*diet*level*size combination) each being fed to 10 individuals (Table S24). To calculate this ration we used monthly monitoring of sentinel fish. Each month, we identified two individuals for each sex*diet*size combination from the 100% feeding level (e.g. 100% fed large male on the 10.2:1 diet, see supplementary table S24). Food was added to the tank in small increments and feeding behaviour was observed. If all the food was consumed another portion of food was added to the tank. This continued until satiation. This was done at both feeding periods for a single day. We then took an average of the amount of food eaten across the four feeding assessments (two individuals with two assessments each), and this ration was fed to all individuals on that treatment for the next month. The individuals used to determine food ration were the two median sized fish of that treatment.

**Data Collection**

**Mortality**

Fish were checked twice daily for mortality and date of death was recorded. For welfare reasons and to comply with home office regulations in the UK, any individuals showing signs of ill health were monitored closely and, if symptoms persisted for two consecutive days, were humanely sacrificed via overdose of tricaine mesylate (MS222) and physical destruction of the brain. Symptoms of ill health were typically gulping at the surface or bottom of the tank, inability to maintain an upright position whilst swimming or females being egg bound. Fish showing these symptoms typically do not recover (Walling personal observation) and so we do not feel this protocol biased data on survival in any way. On day 749 of the experiment only 53 individuals remained alive (46 males and 7 females). For logistical reasons, these individuals were culled using standard approved procedures in the UK and analysed as censored data points.

**Male Reproductive Investment**

In the wild, sticklebacks typically undergo a single breeding season per year, commencing during the early spring and lasting through to late summer or early autumn (Wootton 1984). However, under laboratory conditions, sticklebacks can undergo a second breeding season, though typically this involves a small number of individuals and limited reproduction (e.g. Inness & Metcalfe 2008). A number of fish survived to a second breeding season in our experiment and although we recorded any reproductive activity, not enough individuals attempted to reproduce to allow meaningful statistical analysis. Therefore, all reproductive data presented in the main text are from the first season, which represents a good proxy for lifetime reproductive investment.

*Nesting Behaviour*

In the wild, male three-spine sticklebacks construct nests from filamentous algae and sediment during the breeding season, in which females lay eggs (Wootton 1984). Once a male developed nuptial colour, they were provided with standard nesting material, consisting of approximately 200 6cm long black cotton threads and sand (Barber *et al.* 2001). Males were then stimulated to build nests by presenting them with an image of a sexually mature (gravid) female for 5 minutes, once per day. Nests were checked daily and the time until nest construction started and time until construction was completed were recorded. Males were given 2 weeks at each of these stages of nest construction (i.e. two weeks to start a nest, 2 weeks to complete a nest and 2 weeks with a completed nest), at the end of the two week period the nesting material was removed and replaced with fresh material. Nesting assessment continued until a male failed to attempt nest construction on three successive occasions, after which no further nesting material was provided.

*Courtship*

While nesting, we assayed males for common reproductive behaviours (Wootton 1984): defence of the breeding territory and courtship. Male courtship can be stimulated by exposing the male to an image of a sexually mature female (Moatt and Walling personal observation). Male courtship behaviour comprises a number of discrete steps (see Wootton 1984), the two most recognisable steps being the ‘zig-zag dance’ towards the female, during which the male rapidly swims from side to side, followed by the male ‘leading’ the female to the nest and swimming through the nest entrance (Wootton 1984).We assayed courtship ability 7 days after a nest was judged to be completed. Males were assessed for 5 minutes, during which an image of a gravid female was attached to the front of the tank. During this time we recorded: reaction time (i.e. the time from the image being presented until the male attempted courtship), total time spent courting the female, number of zig-zag dances and number of leads. This was repeated for all nests that were completed by a male. For final analysis we analysed the average reaction time (s), total time spent courting (s), total number of zigzag dances and the total number of leads across all trials for each individual male. In the main paper we report the results from analysis of total time spent courting, as the effect of macronutrients on courtship were consistent across all measures of courtship.

*Territory Defence*

Males display aggressive behaviour towards any red object (Wootton 1984), therefore we stimulated aggressive displays by suspending a red pen lid in the male’s home tank. Males were exposed to the red object once per nest (within 7 days of construction beginning) for a period of 5 minutes and this was repeated for every nest a male started to construct. During the observation period we recorded: reaction time, total time displaying aggressive behaviour, number of aggressive swims and number of biting attempts. For final analysis we explored: the average reaction time, total time displaying aggressive behaviour and the total number of aggressive swims across all trials for each individual male. Results from these analyses are qualitatively similar to those from analyses of courtship behaviour and so are only presented in the supplement.

*Nuptial Colouration*

In addition to the above measures, we also measured the age-specific investment in nuptial colouration by male sticklebacks (see below). During the breeding season, male three-spine sticklebacks develop red nuptial colouration (Wootton 1984). From the start of the male breeding season (defined as > 20% of males having developed nuptial colouration), males were photographed monthly using standard procedures (see Frischknecht 1993; Braithwaite & Barber 2000 ; Barber *et al.* 2001). Briefly, fish were removed from their home tank and immediately placed in a glass-sided photographing chamber filled with water. Males were placed so their right side faced towards the camera and were temporarily fixed in place with a piece of damp sponge. The photographing chamber was then placed onto a small viewing stage and an image taken under standard light conditions (see below). Immediately after the picture was taken, males were returned to their home tank. The whole process from removal to returning a male its home tank took approximately 60s and was designed to minimise the stress experienced by the males. This procedure was performed approximately once a month until the breeding season was deemed to have ended (when less than 20% of males were displaying colour). Light conditions were standardised by the use of two lamps containing broad spectrum daylight bulbs angled at 45^o^ towards the viewing stage, with no flash used on the camera. Photographs were taken with a Pentax K*_r_* digital camera (F2.8, shutter speed 1/125), fitted with a Tamron 90mm macro lens fixed in position directly in front of the viewing stage. The relative positions of the viewing stage, camera and two lamps remained constant throughout the experiment. All photographs were taken alongside a scale bar and white, grey and black colour standards.

Photographs were analysed using the software ImageJ. Briefly, the white standard was analysed to give standard values for red, green and blue light. The fish was then highlighted using the polygon selection tool ensuring just the main body of the fish was included. The intensity and area of red colouration were recorded and the process was then repeated for blue colour. Prior to analysis colour intensity was standardised by dividing by values obtained from a white colour standard included in all photographs (e.g. red intensity = red _fish_ / white _standard_).

Unlike other measures of reproduction (e.g. courtship) there was no obvious way of summing measures of colouration to create a cumulative (lifetime) measure. We therefore investigated age specific investment in nuptial colouration for males. This analysis was carried in the same manner as the age-specific analysis of courtship and egg laying (see methods in main text and below) including the same fixed and random effects. However, as all males were photographed at the same time points, we did not include age of first reproductive event as a covariate. Because some males die during the breeding season, we did include age of last record of colouration to account for selective disappearance from the population. Analysis of age specific investment in male nuptial colouration was qualitatively similar to that of age-specific courtship investment and so presentation of results is limited to the supplementary materials.

**Female Reproductive Investment**

Females are capable of producing multiple egg clutches per breeding season (Wootton 1984) and can be stripped of eggs without causing any ill effects (Barber & Arnott 2000). Females were checked daily to see if they were gravid (indicated by a swollen and distended abdomen (Barber & Arnott 2000). Gravid females were removed from the tank, quickly dried, weighed and the eggs stripped and placed into a petri dish. Egg stripping involved gently running a finger down the side of the fish towards the tail, which encouraged the expulsion of the egg sack. This was repeated on both sides of the fish, ensuring all eggs were expelled. The fish was then quickly reweighed and returned to their original tank. The whole process from removal to returning to home tank took approximately 60s. The eggs were then spread into a monolayer using fine forceps and a paintbrush and counted twice to ensure accuracy. The number of eggs was taken as the average between the two numbers (rounded to the nearest whole number). Clutch mass was calculated as the difference in pre- and post- stripping weight of the female. Fish were stripped whenever they became gravid with the shortest interval between two egg strips being 3 days. We therefore quantified: the total number of eggs produced by each female, the total number of egg clutches produced by a female and the average number of eggs per clutch. Thus we can distinguish whether any differences in lifetime egg production are due to females producing more clutches, more eggs per clutch or both. Occasionally, a female would lay eggs naturally before they could be manually stripped (N=210 out of 2766 clutches). To allow us to get a measure of total number of eggs produced, a natural laying event was given an average egg score (mean number of eggs per clutch for that female).

**Weighing Procedure**

Fish size is expected to correlate with fitness, as bigger females can produce more eggs and bigger males are more competitive (Wootton, 1973; Wootton, 1984). Therefore fish were monitored for growth throughout life. Prior to the start of dietary manipulations, fish were weighed (g) and length taken (mm). We used a standard weighing procedure where fish are removed from their tank, dried and weighed on a balance (± 0.01g). Once weighed the length (± 0.5mm) of the fish was measured from head to tail fork and the fish was returned to its original tank, with the whole process taking less than 60s. Fish were weighed approximately every 1-2 months from the start of the experiment (November 2014) until the end (December, 2016; Table S26). Partial data for the August 2015 weighing was lost, however this was a period of little to no growth. For the analysis presented below, we only look at growth over the first 10 measurements (November 2014 – May 2016), as from July 2016 onwards there were few individuals alive, resulting in lack of statistical power (see Table S26).

**Body Condition**

A common measure of overall health in fish is body condition index, a measure of the weight of an individual relative to its size (length). Here, we calculated body condition using residuals from an analysis of the length-weight relationship (see Bentley & Schindler 2013; Moatt *et al.* 2017):

$$Condition Index=log\left( Weight \right)-\log\left( a \right)-b\log(Length)$$

With the slope (b) and intercept (a) taken from a model of the log of weight against the log of length for all fish measured in this study (Bentley & Schindler 2013; Moatt *et al.* 2017). A negative value indicates an individual weighing less than average for its length, whilst a positive value suggests an individual weighing more than average for its length. Condition index was calculated for each time period independently, meaning a value of 0 is the average condition for each time period. Both sexes were included in the intercept and slope calculation, making the condition index score relative to the whole population.

**Statistical Analysis**

All analyses were carried out in R (v3.4.0, R core team, 2017). We used a response-surface approach (Lande & Arnold 1983) to estimate the linear and non-linear effects (quadratic and correlation) of protein and lipid intake and the interaction between them on our response variables (Lee *et al.* 2008; Maklakov *et al.* 2008; Fanson *et al.* 2009; Maklakov *et al.* 2009; Solon-Biet *et al.* 2014; Jensen *et al.* 2015 and see below). As recommended (Lande & Arnold 1983) estimates of linear terms were taken from a model containing only linear terms whereas estimates of non-linear terms were taken from a model including linear and non-linear terms. For all analyses, protein and lipid intakes were standardised to a mean of zero and a standard deviation of one to avoid issues of scale differences when fitting quadratic terms. For all traits we performed separate analyses for each sex. To test for sex-specific effects of macronutrients we then combined the data and performed a full analysis with sex interacted with protein and lipid. For models comparing reproductive investment between the sexes, response variables were also standardised to a mean of zero and a standard deviation of one to facilitate comparison. Below, we present the model formula for all of the models fitted. The R script for all models is also stored on the dryad repository (doi:10.5061/dryad.g12p0j2). Nutritional landscapes were visualised using thin-plate splines from the package *fields*.

Survival was analysed using generalised linear mixed models (GLME) in the package *Lme4* (Bates *et al.* 2015), implementing an event history analysis (e.g. Therneau & Grambsch 1991). Previous similar experiments have analysed lifespan against intake rates measured from a period when growth has ceased and thus intake rates are stable (e.g. Solon-Biet *et al.* 2014). However, sticklebacks have indeterminate growth and thus intake rates change over time, making this approach inappropriate. Event history models allow for time varying covariates. We therefore analyse mortality as a weekly event. Individuals’ were assigned a weekly survival value (0 = survived, 1 = death), which represents the response variable. This was then modelled against the linear and quadratic effects of protein and lipid intake for that week. These models also included initial weight as a continuous covariate to control for differences in mortality due to size that were not determined by diet. Visual inspection of mortality risk revealed clear variation across the study period (Figure S1). We therefore subdivided the experiment into six periods where the mortality risk was noticeably different to assess whether the impact of intake on mortality risk varied across these periods (Figure S1). Models also included experimental week and individual identity as random effects to allow for heterogeneity in mortality risk across time and individuals. The 53 individuals that survived to the end of the experiment were included as censored data points, never having a weekly survival of 1. These models return a per interval probability of death on the logit scale, which we term ‘mortality risk’ throughout.

***Statistical models fitted: Weekly survival (for each sex separately):***

Linear model:

= TP + P + L + BW + (1|ID) + (1|Week) + (1|Tank)

Non-Linear model:

= TP + P + L + BW + P:L + P^2^ + L^2^ + (1|ID) + (1|Week) + (1|Tank)

Interaction model:

= TP + P + L + BW + P:L + P^2^ + L^2^ + TP:P + TP:L + TP:P:L + TP:P^2^ + TP:L^2^ + (1|ID) + (1|Week) + (1|Tank)

***Weekly survival (comparison of sexes):***

Linear model:

= TP + S + P + L + BW + (1|ID) + (1|Week) + (1|Tank)

Non-Linear model:

= TP + P + L + BW + P:L + P^2^ + L^2^ + (1|ID) + (1|Week) + (1|Tank)

Interaction model (minimal):

= TP + P + L + BW + L^2^ + S:P + S:L + S:L^2^ + TP:L^2^ + TP:S (1|ID) + (1|Week) + (1|Tank)

*TP = time period, P = protein, L = lipid, BW = body weight, S = sex*

Measures of total reproductive investment (total time courting, total time defending breeding territory and total number of eggs produced) were analysed using linear mixed effects models (LME). The linear and quadratic effects of protein and lipid were included as continuous covariates, with shared tank and family group included as random effects. As intake rates were stable throughout the breeding season, we analysed the average daily intake (gday^-1^) of protein and lipid across the course of the breeding season.

***Statistical models fitted: Reproduction (separate sex analysis):***

Linear model:

= P + L + (1|Tank) + (1|Family)

Non-Linear model:

= P + L + P:L + P^2^ + L^2^ + (1|Tank) + (1|Family)

***Weekly survival (comparison of sexes):***

Linear model:

= S + P + L + (1|Tank) + (1|Family)

Interaction model:

= S + P + L + P:L + P^2^ + L^2^ + S:P + S:L + S: P^2^ + S:L^2^ + S:P:L + (1|Tank) + (1|Family)

*P = protein, L = lipid, S = sex*

As we monitored both male and female reproduction at multiple time points throughout the breeding season (see above), we also investigated reproductive senescence in both sexes. We used LME models to explore the effects of protein and lipid intake on age-specific reproduction in both sexes (Maklakov *et al.* 2009; Jensen *et al.* 2015). We analysed the number of eggs produced for females and the time courting for males at each time point. We fitted both the linear and non-linear effects of age, protein intake and lipid intake, as well as age of first reproductive event and age of last reproductive event, with individual ID included as a random effect. Including age of first and last reproductive event in the models accounts for within and between individual variation in age-specific reproduction (van de Pol & Verhulst 2006). Previous work used lifespan rather than age of last reproductive event (see Maklakov *et al.* 2009; Jensen *et al.* 2015). However, in our experiment a large number of individuals survived long after they stopped reproducing, making age at last reproduction a more appropriate measure. Estimates for the linear effects of protein and lipid were taken from a model including both the linear and non-linear effect of age, as the effect of age was strongly non-linear.

***Statistical models fitted: Reproductive Senescence (separate sex analysis):***

Linear model:

= A + A^2^ + F + La + P + L + (1|ID) + (1|Tank) + (1|Family)

Non-Linear model:

= A + A^2^ + F + F^2^ + La + La^2^ + P + L + P^2^ + L^2^ + P:L + (1|ID) + (1|Tank) + (1|Family)

Interaction model males (minimal):

= A + A^2^ + F + F^2^ + La + P + L + A:P + A:L + P:L + (1|ID) + (1|Tank) + (1|Family)

Interaction model females (minimal):

= A + A^2^ + F + La + P + L + P^2^ + L^2^ + P:L + A:P + A:L + P:L + A:F + A:La + (1|ID) + (1|Tank) + (1|Family)

***Reproductive Senescence (comparison of sexes):***

Linear model:

= S + A + A^2^ + F + La + P + L + (1|ID) + (1|Tank) + (1|Family)

Interaction model:

= S + A + A^2^ + F + F^2^ + La + La^2^ + P + L + L^2^ + S:A^2^ + S:P + S:L + A:P + A:L + S:A:P + S:A:L + (1|Tank) + (1|Family)

*A = age, F = age of first reproduction, La = age of last reproduction, P = protein, L = lipid, S = sex*

Weight, length and body condition were analysed through general linear mixed model using ASReml-R software (v3.0, Gilmour *et al.* 2009). For initial differences across treatments, response variables were modelled against Diet and Restriction Level (treatment) fitted as factors. To test for initial differences between the sexes, a separate analysis was performed with sex included as factor. When testing the effect of macronutrient intake, protein and lipid intakes were calculated as the average daily intake (gday^-1^) for the period between each measurement (i.e. the average daily intake for the period from time period 1 to time period 2), with the linear and non-linear effects of protein and lipid included as continuous covariates. All models included time period as a factor, with protein and lipid being interacted with time period to test for changing effects over time. For models exploring differences between the sexes, sex was included as a factor and then interacted with each variable. If there was evidence of an interaction between sex and a specific dietary component, the three way interaction between time period, sex and the macronutrient was included to check for changing effects over time. We included a first order autoregressive function on the residual covariance matrix to allow different residual variance at each time period and to control for autocorrelation as a result of repeated measures on the same individual.

***Statistical models fitted: Length (separate sex analysis):***

Linear model:

= TP + P + L + (TP | ID)

Non-Linear model:

= TP + P + L + P:L + P^2^ + L^2^ + (TP | ID)

Interaction model males (minimal):

= TP + P + L + P:L + P^2^ + L^2^ + TP:P + TP:L + (TP | ID)

Interaction model females (minimal):

= TP + P + L + P:L + P^2^ + L^2^ + TP:L + (TP | ID)

***Length (comparison of sexes):***

Linear model:

= S + TP + P + L + (TP | ID)

Interaction model:

= TP + P + L + P:L + P^2^ + L^2^ + TP:P + TP:L + TP:P^2^ + TP:L^2^ + TP:P:L + S:P + S:P^2^ + S:L + S:L^2^ + S:P:L + (TP | ID)

*TP = time period, P = protein, L = lipid, S = sex*

***Body Condition (separate sex analysis):***

Linear model:

= TP + P + L + (TP | ID)

Non-Linear model:

= TP + P + L + P:L + P^2^ + L^2^ + (TP | ID)

Interaction model males:

= TP + P + L + P^2^ + L^2^ + TP:P + TP:L + TP:P^2^ + TP:L^2^ + (TP | ID)

Interaction model females:

= TP + P + L + P^2^ + L^2^ + P:L + TP:P + TP:L + T:P^2^ + TP:L^2^ + TP:P:L + (TP | ID)

***Body Condition (comparison of sexes):***

Linear model:

= S + TP + P + L + (TP | ID)

Interaction model (minimal):

= S + TP + P + L + L^2^ + S:T + TP:L + S:P + S:L (TP | ID)

*TP = time period, P = protein, L = lipid, S = sex*

Intakes were analysed using an adaptation of a previous method (see Fanson *et al.* 2009; Solon-Biet *et al.* 2014). Intakes rates were calculated as an average for each sentinel fish (see supplementary methods) for a period of stable intakes from August 2015 to February 2016 (195 days). As only these sentinel fish have natural intake rates for the five diets, we have insufficient data to test the independent effect of protein and lipid content of the diet on intake. Therefore we analyse intake rates using a general linear model with Diet and Size, fitted as factors, and the interaction between them for each sex. As there was no evidence of any effect of sex on intake rates, we did not fit the interaction between sex and any other covariates.

*Intakes (n.b. sex was non-significant so dropped from models)*

= Diet + Size + Diet:Size

Dialog S2: Interpretation of Response Surfaces

As in other studies using the GF (e.g. Maklakov *et al.* 2008; Jensen *et al.* 2015), we present the effects of macronutrient intake on specific traits using non-parametric thin-plate splines to generate a response surface. In figures 1 and 2, specific macronutrient intakes are presented on the x-axis (protein) and y-axis (lipid). The trait of interest is then plotted on the z-axis, with the specific trait values indicated on the contour lines. Colours aid in the visualisation of the change in trait value, with trait values varying from low (blue) to high (red). Curvature of the contour lines indicates non-linear (i.e. quadratic) effects of macronutrients.

The five solid lines from the origin are diet rails, representing the 5 diets used in this experiment (see Table 1 and Table S25). The dashed diagonal lines indicate isocaloric intakes on the different diet rails. These isocaloric lines are to aid interpretation only and do not represent the restriction levels used in this study.

When exploring the effect of time (e.g. Age – Figure 3 or Time Period – Figures 4 & 5) we fit time on the x-axis and the specific macronutrient on the y-axis, with separate panels for each macronutrient (e.g. Figure 4, see also Maklakov *et al*. 2009; Jensen *et al*. 2015). The surface is as above, with contours and colours indicating a change in trait (blue – low, red – high). The angle and curvature of the contours at any particular point on the x-axis represents the effect of the intake of each macronutrient at that time (Age (Figure 3) or Time point (Figures 4 and 5)). Following the change in contours along the x‑axis allows visualisation of how the trait changes with time and how the effect of macronutrient changes with time (e.g. figure 4). To aid interpretation of the combined effects of protein and lipid in figures 4 and 5, we have included independent plots for each time period in the supplement (figures S5 and S8 respectively).

Dialog S3: Supplementary Results and Discussion

**Nuptial Colour**

There was a significant non-linear effect of age on male nuptial colour, with red intensity increasing to a peak at the height of the breeding season then declining towards the end (Fig S10, Table S27). Red intensity highest at intermediate intakes (Fig S10, Table S27), however there was no evidence of an interaction between lipid and age, suggesting that lipid intake did not alter the rate of senescence in nuptial colouration (Fig S10, Table S27). Protein intake had no effect on red intensity or any interaction with age (Fig S10, Table S27). However, there was a positive interaction between protein and lipid intake, suggesting the beneficial effect of lipid was stronger at higher protein intakes (Table S27). Finally, there was a marginally non-significant positive linear effect of age of last measurement, suggesting longer living males had slightly higher red intensity (Table S27).

**Female Reproductive Senescence**

There was a negative linear effect of age of first reproductive attempt on subsequent reproduction, meaning those individuals starting to reproduce later in life produced smaller clutches (Table S11). There was also a significant effect of age of last reproductive attempt on clutch size, suggesting that individuals reproducing later in life overall produced larger clutches (Table S11). There was a positive interaction between age of first reproduction with age, suggesting that those individuals starting reproduction early in life produced larger clutches but suffered a faster rate of reproductive senescence in later life (Fig S4 Table S11). Similarly, there was an interaction between age of last reproduction with age, which showed that individuals reproducing later in life have a faster rate of increasing clutch size in early life, however, they also suffer a greater decline in reproduction late in their reproductive life (Fig S4 Table S11).

**Sex Differences in Growth and Condition (Health)**

There were significant differences between the sexes in initial size with females being longer (mean length (mm) ± s.e.: males = 34.09 ± 0.24; females = 35.50 ± 0.22; Fig S7) and heavier (mean weight (g) ± s.e.: males = 0.46 ± 0.01; females = 0.50 ± 0.01; Fig S7) than males. These difference remained throughout the course of the experiment and increased over time (Tables S17 and S18; Fig S7).

Unlike size, there were no differences in initial condition between the sexes (Fig S7). There were significant differences between the sexes in final condition (Fig S7, Table S21), with males being in better condition than females (mean condition ± s.e.: Males = 0.016 ± 0.012; Females = ‑0.029 ± 0.015; Fig S7). Given the sex specific effects of macronutrient intake on condition, we suggest this is due to differential utilisation of ingested macronutrients between the sexes. As male three-spine sticklebacks have higher adiposity than females (Moatt *et al.* 2017) we suggest males utilise less resources for growth, ensuring high energy reserves and better overall condition. In contrast, females invest heavily in growth, growing both longer and heavier than males, but with fewer energy stores (Moatt *et al.* 2017). This difference may be due to different reproductive behaviours exhibited by the sexes. For females, there is a well-known association between size and egg production (Wootton 1973), with larger females producing larger clutches and thus achieving higher fitness. However, males may require greater energy reserves than females, owing to their energetically costly and time consuming reproductive behaviours (such as egg fanning) preventing males form foraging fully during the breeding season (Rohwer 1978; Moatt *et al.* 2017). Thus, a male who is in better condition, with higher fat deposits, needs to forage less, can invest more in reproduction, and thus achieves higher fitness.

References

Barber I, Arnott SA (2000). Split-clutch IVF: a technique to examine indirect fitness consequences of mate preferences in sticklebacks. *Behaviour*. **137**, 1129-1140.

Barber I, Nairn D, Huntingford FA (2001). Nests as ornaments: revealing construction by male sticklebacks. *Behavioral Ecology*. **12**, 390-396.

Bates D, Mächler M, Bolker B, Walker S (2015). Fitting linear mixed-effects models using lme4. *Journal of Statisitcal Software.* **67**, 48.

Bentley KT, Schindler DE (2013). Body condition correlates with instantaneous growth in stream-dwelling rainbow trout and arctic grayling. *Transactions of the American Fisheries Society*. **142**, 747-755.

Braithwaite VA, Barber I (2000). Limitations to colour-based sexual preferences in three-spined sticklebacks (*Gasterosteus aculeatus*). *Behav Ecol Sociobiol*. **47**, 413-416.

Fanson BG, Weldon CW, Pérez-Staples D, Simpson SJ, Taylor PW (2009). Nutrients, not caloric restriction, extend lifespan in Queensland fruit flies (*Bactrocera tryoni*). *Aging Cell*. **8**, 514-523.

Frischknecht M (1993). The breeding colouration of male three-spined sticklebacks (*Gasterosteus aculeatus*) as an indicator of energy investment in vigour. *Evolutionary Ecology*. **7**, 439-450.

Gilmour AR, Gogel B, Cullis B, Thompson R, Butler D (2009). ASReml user guide release 3.0. *VSN International Ltd, Hemel Hempstead, UK*.

Inness CL, Metcalfe NB (2008). The impact of dietary restriction, intermittent feeding and compensatory growth on reproductive investment and lifespan in a short-lived fish. *Proceedings of the Royal Society B:Biological Sciences*. **275**, 1703-1708.

Jensen K, McClure C, Priest NK, Hunt J (2015). Sex‐specific effects of protein and carbohydrate intake on reproduction but not lifespan in *Drosophila melanogaster.* *Aging cell*. **14**, 605-615.

Lande R, Arnold SJ (1983). The measurement of selection on correlated characters. *Evolution*, **37**, 1210-1226.

Lee KP, Simpson SJ, Clissold FJ, Brooks R, Ballard JW, Taylor PW, Soran N, Raubenheimer D (2008). Lifespan and reproduction in *Drosophila*: New insights from nutritional geometry. *PNAS*. **105**, 2498-2503.

Maklakov AA, Hall MD, Simpson SJ, Dessmann J, Clissold FJ, Zajitschek F, Lailvaux SP, Raubenheimer D, Bonduriansky R, Brooks RC (2009). Sex differences in nutrient-dependent reproductive ageing. *Aging Cell*. **8**, 324-330.

Maklakov AA, Simpson SJ, Zajitschek F, Hall MD, Dessmann J, Clissold F, Raubenheimer D, Bonduriansky R, Brooks RC (2008). Sex-specific fitness effects of nutrient intake on reproduction and lifespan. *Current Biology* **18**, 1062-1066.

Moatt JP, Hambly C, Heap E, Kramer A, Moon F, Speakman JR, Walling CA (2017). Body macronutrient composition is predicted by lipid and not protein content of the diet. *Ecology and Evolution*. **7**, 10056-10065.

Rohwer S (1978). Parent cannibalism of offspring and egg raiding as a courtship strategy. *The American Naturalist*. **112**, 429-440.

Ruohonen K, Koskela J, Vielma J, Kettunen J (2003). Optimal diet composition for European whitefish (*Coregonus lavaretus*): analysis of growth and nutrient utilisation in mixture model trials. *Aquaculture*. **225**, 27-39.

Ruohonen K, Simpson SJ, Raubenheimer D (2007). A new approach to diet optimisation: A re-analysis using European whitefish (*Coregonus lavaretus*). *Aquaculture*. **267**, 147-156.

Solon-Biet SM, McMahon AC, Ballard JWO, Ruohonen K, Wu LE, Cogger VC, Warren A, Huang X, Pichaud N, Melvin RG (2014). The ratio of macronutrients, not caloric intake, dictates cardiometabolic health, aging, and longevity in *ad libitum*-fed mice. *Cell Metabolism*. **19**, 418-430.

Terzibasi E, Lefrançois C, Domenici P, Hartmann N, Graf M, Cellerino A (2009). Effects of dietary restriction on mortality and age‐related phenotypes in the short‐lived fish *Nothobranchius furzeri*. *Aging Cell*. **8**, 88-99.

Therneau TM, Grambsch PM (1991). *Modeling survival data: extending the cox model.* Springer-Verlag, New York.

van de Pol M, Verhulst S (2006). Age-dependent traits: a new statistical model to separate within-and between-individual effects. *The American Naturalist*. **167**, 766-773.

Wootton RJ (1973). Fecundity of the three-spined stickleback, *Gasterosteus aculeatus*. *Journal of Fish Biology*. **5**, 683-688.

Wootton RJ (1984). *A functional biology of sticklebacks*: University of California Press.

Supplementary Tables

**Table S1. Outputs from event history models (binomial GLME) exploring Male mortality risk.**

|  | **Estimate (± s.e.)** | **χ^2^** | ***p*** |
| --- | --- | --- | --- |
| **Linear Model** | | | |
| *intercept* | -3.999 (0.349) |  |  |
| Time Period 2 | -1.076 (0.299) |  |  |
| Time Period 3 | 0.561 (0.319) |  |  |
| Time Period 4 | -0.334 (0.356) |  |  |
| Time Period 5 | 1.813 (0.399) |  |  |
| Time Period 6 | 1.639 (0.520) | 89.15 | < 0.001 |
| Protein | 0.223 (0.133) | 2.87 | 0.090 |
| Lipid | -0.396 (0.136) | 9.47 | 0.002 |
| Initial Weight | -1.044 (0.597) | 3.29 | 0.070 |
| **Non-Linear Model** | | | |
| Protein^2^ | 1.095 (0.738) | 2.20 | 0.138 |
| Lipid^2^ | 1.379 (0.657) | 4.32 | 0.038 |
| Protein*Lipid | -0.321 (0.295) | 1.17 | 0.279 |
| **Interaction Model** | | | |
| *intercept* | -4.684 (0.311) |  |  |
| Time Period 2 | -0.847 (0.344) |  |  |
| Time Period 3 | 0.812 (0.349) |  |  |
| Time Period 4 | -0.162 (0.385) |  |  |
| Time Period 5 | 2.444 (0.741) |  |  |
| Time Period 6 | 1.159 (1.167) | 88.16 | < 0.001 |
| Lipid | -1.590 (0.631) | 10.25 | 0.001 |
| Lipid^2^ | 1.212 (0.794) | 7.07 | 0.008 |
| Time Period 2*Lipid | -0.283 (0.965) |  |  |
| Time Period 3*Lipid | -0.146 (0.863) |  |  |
| Time Period 4*Lipid | 0.236 (0.875) |  |  |
| Time Period 5*Lipid | 0.325 (1.172) |  |  |
| Time Period 6*Lipid | 2.987 (1.927) | 7.78 | 0.168 |
| Time Period 2*Lipid^2^ | 0.397 (1.060) |  |  |
| Time Period 3*Lipid^2^ | 0.241 (0.959) |  |  |
| Time Period 4*Lipid^2^ | -0.006 (0.989) |  |  |
| Time Period 5*Lipid^2^ | 0.892 (2.111) |  |  |
| Time Period 6*Lipid^2^ | -3.741 (3.570) | 1.79 | 0.877 |

Linear model contains only the linear terms, Non-linear model contains all linear terms and the listed non-linear and interaction effects and the Interaction model contains main effects that were significant in previous models (linear and non-linear) and their interactions.

**Table S2.** Outputs from Cox Proportional Hazards Models exploring the effect of Diet, Food Level and Sex on survival (weeks).

|  | **Χ^2^** | **DF** | ***p*** |
| --- | --- | --- | --- |
| **Males** | | | |
| Diet | 12.75 | 4 | 0.013 |
| Food Level | 0.58 | 2 | 0.747 |
| Diet*Food Level | 6.06 | 8 | 0.640 |
| **Females** | | | |
| Diet | 9.89 | 4 | 0.042 |
| Food Level | 0.90 | 2 | 0.639 |
| Diet*Food Level | 9.68 | 8 | 0.289 |
| **Combined** | | | |
| Diet | 11.19 | 4 | 0.024 |
| Food Level | 1.04 | 2 | 0.593 |
| Sex | 33.93 | 1 | < 0.001 |
| Diet*Food Level | 5.68 | 8 | 0.683 |
| Diet*Sex | 6.96 | 4 | 0.138 |

**Table S3.** **Outputs from event history models (binomial GLME) exploring Female mortality risk.**

|  | **Estimate (± s.e.)** | **χ^2^** | ***p*** |
| --- | --- | --- | --- |
| **Linear Model** | | | |
| *intercept* | -5.040 (0.408) |  |  |
| Time Period 2 | 0.034 (0.341) |  |  |
| Time Period 3 | 1.736 (0.381) |  |  |
| Time Period 4 | 1.185 (0.444) |  |  |
| Time Period 5 | 3.058 (0.597) |  |  |
| Time Period 6 | 3.333 (0.819) | 78.38 | < 0.001 |
| Protein | 0.032 (0.094) | 0.12 | 0.733 |
| Lipid | -0.002 (0.089) | < 0.00 | 0.981 |
| Initial Weight | -0.066 (0.499) | 0.02 | 0.894 |
| **Non-Linear Model** | | | |
| Protein^2^ | -0.243 (0.435) | 0.31 | 0.576 |
| Lipid^2^ | 0.255 (0.453) | 0.31 | 0.575 |
| Protein*Lipid | -0.032 (0.160) | 0.04 | 0.843 |
| **Interaction Model** | | | |
| *intercept* | -5.927 (0.617) |  |  |
| Time Period 2 | 0.814 (0.620) |  |  |
| Time Period 3 | 2.594 (0.643) |  |  |
| Time Period 4 | 2.074 (0.693) |  |  |
| Time Period 5 | 4.480 (0.880) |  |  |
| Time Period 6 | 1.311 (1.734) | 78.36 | < 0.001 |
| Protein | -1.466 (0.722) | 0.14 | 0.707 |
| Lipid | 0.810 (0.444) | 0.03 | 0.866 |
| Time Period 2*Lipid | -1.036 (0.479) |  |  |
| Time Period 3*Lipid | -0.739 (0.456) |  |  |
| Time Period 4*Lipid | -0.978 (0.470) |  |  |
| Time Period 5*Lipid | -0.498 (0.592) |  |  |
| Time Period 6*Lipid | -0.492 (0.997) | 7.15 | 0.210 |
| Time Period 2*Protein | 1.966 (0.751) |  |  |
| Time Period 3*Protein | 1.454 (0.730) |  |  |
| Time Period 4*Protein | 1.420 (0.732) |  |  |
| Time Period 5*Protein | 1.921 (0.856) |  |  |
| Time Period 6*Protein | -1.736 (1.795) | 16.16 | 0.006 |

Linear model contains only the linear terms, Non-linear model contains all linear terms and the listed non-linear and interaction effects and the Interaction model contains main effects that were significant in previous models (linear and non-linear) and their interactions.

**Table S4.** **Outputs from LME models exploring the effect of macronutrient intake on courtship investment (total time courting (s)).**

|  | **Estimate (± s.e.)** | **χ^2^** | ***p*** |
| --- | --- | --- | --- |
| **Linear Model** | | | |
| *intercept* | 434.58 (51.92) |  |  |
| Protein | 86.05 (32.90) | 6.72 | 0.010 |
| Lipid | -28.50 (33.98) | 0.71 | 0.397 |
| **Non-Linear Model** | | | |
| Protein^2^ | -233.94 (212.61) | 1.22 | 0.269 |
| Lipid^2^ | -347.51 (183.45) | 3.52 | 0.061 |
| Protein*Lipid | 61.25 (70.14) | 0.76 | 0.383 |

**Table S5.** **Outputs from LME and GLME models exploring the linear and non-linear effects of protein and lipid on various measures of male courtship effort.**

|  | **Estimate (± s.e.)** | **χ^2^** | ***p*** |
| --- | --- | --- | --- |
| **Reaction Time** | | | |
| Protein | -10.63 (6.59) | 2.61 | 0.106 |
| Lipid | 11.63 (6.79) | 2.94 | 0.087 |
| Protein^2^ | 9.80 (42.74) | 0.05 | 0.817 |
| Lipid^2^ | 25.93 (36.94) | 0.04 | 0.483 |
| Protein * Lipid | 5.42 (14.04) | 0.15 | 0.700 |
| **ZigZag dances** | | | |
| Protein | 0.13 (0.05) | 7.30 | 0.006 |
| Lipid | -0.02 (0.05) | 0.08 | 0.774 |
| Protein^2^ | -0.60 (0.31) | 3.76 | 0.376 |
| Lipid^2^ | -0.27 (0.29) | 0.87 | 0.350 |
| Protein * Lipid | 0.18 (0.11) | 2.56 | 0.109 |
| **Leads** | | | |
| Protein | 0.24 (0.03) | 63.03 | < 0.001 |
| Lipid | -0.22 (0.04) | 37.81 | < 0.001 |
| Protein^2^ | -0.36 (0.20) | 3.17 | 0.075 |
| Lipid^2^ | -0.29 (0.19) | 2.30 | 0.130 |
| Protein * Lipid | 0.09 (0.7) | 1.41 | 0.235 |

**Table S6. Outputs from LME and GLME models exploring the linear and non-linear effects of protein and lipid on various measures of male territory defence.**

|  | **Estimate (± s.e.)** | **χ^2^** | ***p*** |
| --- | --- | --- | --- |
| **Time displaying** | | | |
| Protein | 76.95 (29.45) | 6.66 | 0.010 |
| Lipid | -31.97 (30.31) | 1.12 | 0.291 |
| Protein^2^ | -156.34 (192.74) | 0.67 | 0.412 |
| Lipid^2^ | -217.04 (164.76) | 1.72 | 0.189 |
| Protein*Lipid | 56.15 (63.15) | 0.80 | 0.371 |
| **Aggressive displays** | | | |
| Protein | 0.22 (0.04) | 31.19 | < 0.001 |
| Lipid | -0.22 (0.04) | 26.12 | < 0.001 |
| Protein^2^ | 0.16 (0.28) | 0.32 | 0.571 |
| Lipid^2^ | -0.28 (0.26) | 1.08 | 0.299 |
| Protein * Lipid | 0.11 (0.10) | 1.19 | 0.275 |
| **Reaction Time** | | | |
| Protein | -15.70 (5.59) | 7.67 | 0.006 |
| Lipid | 9.90 (5.77) | 2.92 | 0.088 |
| Protein^2^ | -22.64 (36.56) | 0.39 | 0.535 |
| Lipid^2^ | -11.71 (31.28) | 0.14 | 0.707 |
| Protein * Lipid | -3.05 (11.93) | 0.07 | 0.793 |

**Table S7.** **Outputs from GLME models exploring the linear and non-linear effects of protein and lipid on various measures of male nesting behaviour.**

|  | **Estimate (± s.e.)** | **χ^2^** | ***p*** |
| --- | --- | --- | --- |
| **Nesting Attempts** | | | |
| Protein | -0.013 (0.026) | 0.29 | 0.592 |
| Lipid | 0.047 (0.026) | 3.30 | 0.069 |
| Protein^2^ | -0.011 (0.166) | < 0.01 | 0.945 |
| Lipid^2^ | -0.219 (0.142) | 2.41 | 0.121 |
| Protein * Lipid | -0.017 (0.056) | 0.09 | 0.768 |
| **Completed Nests** | | | |
| Protein | 0.026 (0.036) | 0.51 | 0.475 |
| Lipid | 0.059 (0.036) | 2.62 | 0.105 |
| Protein^2^ | 0.014 (0.229) | < 0.01 | 0.952 |
| Lipid^2^ | -0.222 (0.201) | 1.22 | 0.270 |
| Protein * Lipid | -0.001 (0.079) | < 0.00 | 0.994 |

**Table S8.** **Outputs from LME models exploring the effect of macronutrient intake on egg production (total number of eggs).**

|  | **Estimate (± s.e.)** | **χ^2^** | ***p*** |
| --- | --- | --- | --- |
| **Linear Model** | | | |
| *intercept* | 1066.68 (47.67) |  |  |
| Protein | 156.38 (46.12) | 10.93 | < 0.001 |
| Lipid | 58.29 (46.02) | 1.64 | 0.200 |
| **Non-Linear Model** | | | |
| Protein^2^ | 11.95 (274.19) | < 0.00 | 0.960 |
| Lipid^2^ | -906.17(257.99) | 12.27 | < 0.001 |
| Protein*Lipid | 119.07 (87.91) | 1.86 | 0.172 |

**Table S9.** **Outputs from LME models exploring the linear and non-linear effects of protein and lipid on various measures of female egg production.**

|  | **Estimate (± s.e.)** | **χ^2^** | ***p*** |
| --- | --- | --- | --- |
| **Mean Egg Number** | | | |
| Protein | 4.895 (1.907) | 6.56 | 0.010 |
| Lipid | 9.236 (1.897) | 22.91 | < 0.001 |
| Protein^2^ | -10.860 (11.154) | 0.973 | 0.324 |
| Lipid^2^ | -46.642 (10.523) | 18.91 | < 0.001 |
| Protein * Lipid | 7.828 (3.579) | 4.75 | 0.029 |
| **Number of Clutches** | | | |
| Protein | 0.739 (0.350) | 4.22 | 0.040 |
| Lipid | 0.088 (0.349) | 0.07 | 0.789 |
| Protein^2^ | 0.596 (2.113) | 0.09 | 0.769 |
| Lipid^2^ | -4.135 (1.991) | 4.37 | 0.036 |
| Protein * Lipid | 0.231 (0.678) | 0.12 | 0.731 |

**Table S10. Outputs from LME models of reproductive senescence in male courtship.**

|  | **Estimate (± s.e.)** | **χ^2^** | ***p*** |
| --- | --- | --- | --- |
| **Linear Model** | | | |
| *intercept* | -0.039 (0.072) |  |  |
| Age | 1.114 (0.226) |  | < 0.001 |
| Age^2^ | -1.088 (0.226) | 22.95 | < 0.001 |
| Age First | -0.131 (0.043) | 9.39 | 0.002 |
| Age Last | 0.069 (0.043) | 2.60 | 0.107 |
| Protein | 0.118 (0.049) | 5.65 | 0.017 |
| Lipid | -0.082 (0.050) | 2.78 | 0.096 |
| **Non-Linear Model** | | | |
| Age First^2^ | 0.577 (0.297) | 3.83 | 0.050 |
| Age Last^2^ | -0.021 (0.444) | 0.00 | 0.946 |
| Protein^2^ | -0.284 (0.317) | 0.86 | 0.353 |
| Lipid^2^ | -0.266 (0.268) | 1.02 | 0.312 |
| Protein*Lipid | 0.107 (0.102) | 1.19 | 0.275 |
| **Interaction Model** | | | |
| *intercept* | -0.048 (0.074) |  |  |
| Age | 1.168 (0.229) |  |  |
| Age^2^ | -1.135 (0.229) |  |  |
| Age First | -0.741 (0.307) |  |  |
| Protein | 0.126 (0.049) |  |  |
| Lipid | -0.085 (0.050) |  |  |
| Age First^2^ | 0.598 (0.296) |  |  |
| Age*Protein | -0.002 (0.031) | 0.00 | 0.946 |
| Age*Lipid | 0.002 (0.032) | 0.00 | 0.945 |

Courtship = time spent courting (s). All models included the non-linear effect of Age and the *p* value for Age was estimated using the package lmerTest as it is only significant when Age^2^ was included in the model. Linear model contained all linear terms and Age^2^, Non-linear model contains all linear terms and the listed non-linear and interaction effects and the Interaction model contains main effects that were significant in previous models (linear and non-linear) and their interactions.

**Table S11. Outputs from LME models of reproductive senescence in female egg production.**

|  | **Estimate (± s.e.)** | **χ^2^** | ***p*** |
| --- | --- | --- | --- |
| **Linear Model** | | | |
| *Intercept* | -0.042 (0.059) |  |  |
| Age | 1.389 (0.121) | 108.99 | < 0.001 |
| Age^2^ | -1.560 (0.120) | 161.86 | < 0.001 |
| Age First | -0.128 (0.043) | 9.00 | 0.002 |
| Age Last | 0.036 (0.034) | 1.14 | 0.285 |
| Protein | 0.100 (0.054) | 3.42 | 0.064 |
| Lipid | 0.266 (0.053) | 24.57 | < 0.001 |
| **Non-Linear Model** | | | |
| Age First^2^ | 0.054 (0.306) | 0.03 | 0.858 |
| Age Last^2^ | 0.780 (0.323) | 5.75 | 0.016 |
| Protein^2^ | -0.273 (0.285) | 0.96 | 0.327 |
| Lipid^2^ | -1.225 (0.281) | 18.50 | < 0.001 |
| Protein*Lipid | 0.206 (0.097) | 4.59 | 0.032 |
| **Interaction Model** | | | |
| *intercept* | -0.208 (0.084) |  |  |
| Age | 1.857 (0.146) |  |  |
| Age^2^ | -2.038 (0.147) |  |  |
| Age First | -0.126 (0.040) |  |  |
| Age Last | 0.078 (0.037) |  |  |
| Protein | 0.703 (0.278) |  |  |
| Lipid | 1.407 (0.251) |  |  |
| Protein^2^ | -0.646 (0.299) |  |  |
| Lipid^2^ | -1.249 (0.280) |  |  |
| Protein*Lipid | 0.217 (0.097) |  |  |
| Age*Age First | 0.061 (0.017) | 13.14 | < 0.001 |
| Age*Age Last | 0.072 (0.020) | 13.11 | < 0.001 |
| Age*Protein | 0.074 (0.020) | 13.89 | < 0.001 |
| Age*Lipid | 0.028 (0.019) | 2.22 | 0.136 |

Egg production = number of eggs per clutch. All models included the non-linear effect of Age and the *p* value for Age was estimated using the package lmerTest as it is only significant when Age^2^ was included in the model. Linear model contained all linear terms and Age^2^, Non-linear model contains all linear terms and the listed non-linear and interaction effects and the Interaction model contains main effects that were significant in previous models (linear and non-linear) and their interactions.

**Table S12.** **Outputs from LME model exploring sex differences in reproductive senescence.**

|  | **Estimate (± s.e.)** | **χ^2^** | ***p*** |
| --- | --- | --- | --- |
| *intercept* | 0.028 (0.064) |  |  |
| Age | 1.461 (0.129) |  |  |
| Age^2^ | -1.639 (0.129) |  |  |
| Age First | -0.506 (0.218) |  |  |
| Age Last | -0.594 (0.247) |  |  |
| Protein | 0.119 (0.050) |  |  |
| Lipid | 0.733 (0.119) |  |  |
| Sex (male) | 0.002 (0.059) |  |  |
| Age First^2^ | 0.372 (0.209) |  |  |
| Age Last^2^ | 0.662 (0.256) |  |  |
| Lipid^2^ | -0.490 (0.112) |  |  |
| Age*Sex | -0.286 (0.232) | 45.96 | < 0.001 |
| Age^2^*Sex | 0.489 (0.233) | 3.80 | 0.051 |
| Age*Protein | 0.052 (0.020) | 4.44 | 0.35 |
| Age*Lipid | 0.039 (0.020) | 2.55 | 0.110 |
| Protein*Sex | 0.025 (0.072) | 0.04 | 0.838 |
| Lipid*Sex | -0.357 (0.070) | 26.24 | < 0.001 |
| Age*Protein*Sex | -0.052 (0.033) | 2.45 | 0.118 |
| Age*Lipid*Sex | -0.040 (0.034) | 1.42 | 0.234 |

Male values represent total time spent courting (s) and females measures are total egg production. Models contain main effects that split sex models indicated where significant and interactions.

**Table S13.** **Outputs from model of the effect of macronutrient intake on male length.**

|  | **Estimate (± s.e.)** | **F** | **d.f. (1,2)** | ***p*** |
| --- | --- | --- | --- | --- |
| **Linear Model** | | | | |
| *intercept* | 38.840 (0.304) | 59890 | 1, 343.3 | < 0.001 |
| Time Period 2 | 0 (NA) |  |  |  |
| Time Period 3 | 4.513 (0.166) |  |  |  |
| Time Period 4 | 9.150 (0.255) |  |  |  |
| Time Period 5 | 10.484 (0.298) |  |  |  |
| Time Period 6 | 10.445 (0.363) |  |  |  |
| Time Period 7 | 12.026 (0.355) |  |  |  |
| Time Period 8 | 13.462 (0.376) |  |  |  |
| Time Period 9 | 17.805 (0.425) |  |  |  |
| Time Period 10 | 17.849 (0.385) | 334.80 | 8, 844.1 | < 0.001 |
| Protein | -0.727 (0.193) | 14.06 | 1, 660.4 | < 0.001 |
| Lipid | 1.035 (0.203) | 25.86 | 1, 541.9 | < 0.001 |
| **Non-Linear Model** | | | | |
| Protein^2^ | -0.393 (0.092) | 5.30 | 1, 742.3 | 0.022 |
| Lipid^2^ | -0.333 (0.080) | 25.35 | 1, 686.3 | < 0.001 |
| Protein*Lipid | 0.180 (0.038) | 8.54 | 1, 621.6 | 0.004 |
| **Interaction Model** | | | | |
| *intercept* | 37.399 (0.639) | 28060.00 | 1, 580.8 | < 0.001 |
| Time Period 2 | 0 (NA) |  |  |  |
| Time Period 3 | 5.265 (0.408) |  |  |  |
| Time Period 4 | 9.990 (0.551) |  |  |  |
| Time Period 5 | 11.228 (0.576) |  |  |  |
| Time Period 6 | 11.024 (0.614) |  |  |  |
| Time Period 7 | 12.651 (0.599) |  |  |  |
| Time Period 8 | 14.115 (0.612) |  |  |  |
| Time Period 9 | 18.322 (0.646) |  |  |  |
| Time Period 10 | 19.336 (0.734) | 320.10 | 8, 854.9 | < 0.001 |
| Protein | 0.839 (0.968) | 1.35 | 1, 1075.1 | 0.246 |
| Lipid | 3.143 (0.871) | 40.90 | 1, 766.0 | < 0.001 |
| Protein^2^ | -2.159 (0.995) | 4.70 | 1, 856.4 | 0.030 |
| Lipid^2^ | -3.857 (0.833) | 21.43 | 1, 887.2 | < 0.001 |
| Protein*Lipid | 0.873 (0.370) | 5.55 | 1, 816.0 | 0.019 |
| Time Period 2*Protein | 0 (NA) |  |  |  |
| Time Period 3*Protein | 0.122 (0.494) |  |  |  |
| Time Period 4*Protein | -0.129 (0.656) |  |  |  |
| Time Period 5*Protein | 0.076 (0.717) |  |  |  |
| Time Period 6*Protein | 0.345 (0.761) |  |  |  |
| Time Period 7*Protein | 0.097 (0.783) |  |  |  |
| Time Period 8*Protein | 0.417 (0.800) |  |  |  |
| Time Period 9*Protein | 0.343 (0.825) |  |  |  |
| Time Period 10*Protein | 0.346 (0.972) | 1.08 | 8, 822.0 | 0.376 |
| Time Period 2*Lipid | 0 (NA) |  |  |  |
| Time Period 3*Lipid | 1.299 (0.346) |  |  |  |
| Time Period 4*Lipid | 1.553 (0.488) |  |  |  |
| Time Period 5*Lipid | 1.952 (0.541) |  |  |  |
| Time Period 6*Lipid | 2.025 (0.583) |  |  |  |
| Time Period 7*Lipid | 2.163 (0.587) |  |  |  |
| Time Period 8*Lipid | 1.797 (0.607) |  |  |  |
| Time Period 9*Lipid | 2.167 (0.642) |  |  |  |
| Time Period 10*Lipid | 2.667 (0.790) | 3.68 | 8, 820.7 | < 0.001 |

Linear model contained all linear terms, Non-linear model contains all linear terms and the listed non-linear and interaction effects and the Interaction model contains main effects that were significant in previous models (linear and non-linear) and their interactions. Estimates (± s.e.) come from ASreml models, conditional F and *p* obtained through a Wald test.

**Table S14. Outputs from model of the effect of macronutrient intake on male weight.**

|  | **Estimate (± s.e.)** | **F** | **d.f. (1,2)** | ***P*** |
| --- | --- | --- | --- | --- |
| **Linear Model** | | | | |
| (Intercept) | 0.697 (0.021) | 1949 | 1, 431.7 | < 0.001 |
| Time Period 2 | 0 (NA) |  |  |  |
| Time Period 3 | 0.340 (0.012) |  |  |  |
| Time Period 4 | 0.888 (0.023) |  |  |  |
| Time Period 5 | 1.022 (0.027) |  |  |  |
| Time Period 6 | 1.144 (0.033) |  |  |  |
| Time Period 7 | 1.261 (0.032) |  |  |  |
| Time Period 8 | 1.454 (0.036) |  |  |  |
| Time Period 9 | 1.895 (0.043) |  |  |  |
| Time Period 10 | 2.168 (0.043) | 404.50 | 8, 763.6 | < 0.001 |
| Protein | -0.061 (0.022) | 7.99 | 1, 824.5 | 0.005 |
| Lipid | 0.073 (0.022) | 10.68 | 1, 667.2 | 0.001 |
| **Non-Linear Model** | | | | |
| Protein^2^ | -0.393 (0.093) | 17.88 | 1, 1364.8 | < 0.001 |
| Lipid^2^ | -0.334 (0.080) | 17.39 | 1, 1193.0 | < 0.001 |
| Protein*Lipid | 0.181 (0.039) | 21.52 | 1, 1128.7 | < 0.001 |
| **Interaction Model** | | | | |
| *intercept* | 0.980 (0.170) | 2129 | 1, 485.8 | < 0.001 |
| Time Period 2 | 0 (NA) |  |  |  |
| Time Period 3 | -0.107 (0.130) |  |  |  |
| Time Period 4 | 0.461 (0.157) |  |  |  |
| Time Period 5 | 0.609 (0.162) |  |  |  |
| Time Period 6 | 0.742 (0.165) |  |  |  |
| Time Period 7 | 0.822 (0.166) |  |  |  |
| Time Period 8 | 1.013 (0.168) |  |  |  |
| Time Period 9 | 1.444 (0.172) |  |  |  |
| Time Period 10 | 1.249 (0.320) | 476.60 | 8, 756.6 | < 0.001 |
| Protein | -0.282 (0.285) | 4.02 | 1, 1176.6 | 0.045 |
| Lipid | 0.081 (0.214) | 17.34 | 1, 742.9 | < 0.001 |
| Protein^2^ | 0.799 (0.567) | 6.75 | 1, 964.3 | 0.010 |
| Lipid^2^ | -0.034 (0.238) | 23.59 | 1, 1065.7 | < 0.001 |
| Protein*Lipid | 0.025 (0.129) | 13.27 | 1, 942.8 | < 0.001 |
| Time Period 2*Protein | 0 (NA) |  |  |  |
| Time Period 3*Protein | 0.569 (0.184) |  |  |  |
| Time Period 4*Protein | 0.544 (0.232) |  |  |  |
| Time Period 5*Protein | 0.461 (0.250) |  |  |  |
| Time Period 6*Protein | 0.388 (0.271) |  |  |  |
| Time Period 7*Protein | 0.509 (0.272) |  |  |  |
| Time Period 8*Protein | 0.508 (0.283) |  |  |  |
| Time Period 9*Protein | 0.465 (0.301) |  |  |  |
| Time Period 10*Protein | 1.173 (0.604) | 0.65 | 8, 860.7 | 0.739 |
| Time Period 2*Lipid | 0 (NA) |  |  |  |
| Time Period 3*Lipid | 0.576 (0.130) |  |  |  |
| Time Period 4*Lipid | 0.623 (0.171) |  |  |  |
| Time Period 5*Lipid | 0.651 (0.188) |  |  |  |
| Time Period 6*Lipid | 0.686 (0.212) |  |  |  |
| Time Period 7*Lipid | 0.715 (0.210) |  |  |  |
| Time Period 8*Lipid | 0.711 (0.223) |  |  |  |
| Time Period 9*Lipid | 0.789 (0.244) |  |  |  |
| Time Period 10*Lipid | 2.181 (0.532) | 7.73 | 8, 832.2 | < 0.001 |
| Time Period 2*Protein^2^ | 0 (NA) |  |  |  |
| Time Period 3*Protein^2^ | -1.296 (0.401) |  |  |  |
| Time Period 4*Protein^2^ | -1.285 (0.499) |  |  |  |
| Time Period 5*Protein^2^ | -1.172 (0.523) |  |  |  |
| Time Period 6*Protein^2^ | -1.087(0.540) |  |  |  |
| Time Period 7*Protein^2^ | -1.168 (0.546) |  |  |  |
| Time Period 8*Protein^2^ | -1.142 (0.555) |  |  |  |
| Time Period 9*Protein^2^ | -1.096 (0.567) |  |  |  |
| Time Period 10*Protein^2^ | -2.333 (1.052) | 2.41 | 8, 848.1 | 0.014 |
| Time Period 2*Lipid^2^ | 0 (NA) |  |  |  |
| Time Period 3*Lipid^2^ | -0.596 (0.148) |  |  |  |
| Time Period 4*Lipid^2^ | -0.593 (0.186) |  |  |  |
| Time Period 5*Lipid^2^ | -0.544 (0.204) |  |  |  |
| Time Period 6*Lipid^2^ | -0.556 (0.229) |  |  |  |
| Time Period 7*Lipid^2^ | -0.571 (0.229) |  |  |  |
| Time Period 8*Lipid^2^ | -0.553 (0.241) |  |  |  |
| Time Period 9*Lipid^2^ | -0.556 (0.261) |  |  |  |
| Time Period 10*Lipid^2^ | -2.586 (0.675) | 5.19 | 8, 837.3 | < 0.001 |
| Time Period 2*Protein*Lipid | 0 (NA) |  |  |  |
| Time Period 3*Protein*Lipid | 0.205 (0.079) |  |  |  |
| Time Period 4*Protein*Lipid | 0.198 (0.103) |  |  |  |
| Time Period 5*Protein*Lipid | 0.152 (0.111) |  |  |  |
| Time Period 6*Protein*Lipid | 0.119 (0.119) |  |  |  |
| Time Period 7*Protein*Lipid | 0.129 (0.121) |  |  |  |
| Time Period 8*Protein*Lipid | 0.112 (0.126) |  |  |  |
| Time Period 9*Protein*Lipid | 0.084 (0.134) |  |  |  |
| Time Period 10*Protein*Lipid | 0.357 (0.294) | 2.07 | 8, 821 | 0.036 |

Linear model contained all linear terms, Non-linear model contains all linear terms and the listed non-linear and interaction effects and the Interaction model contains main effects that were significant in previous models (linear and non-linear) and their interactions. Estimates (± s.e.) come from ASreml models, conditional F and *p* obtained through a Wald test.

**Table S15.** **Outputs from model of the effect of macronutrient intake on female length.**

|  | **Estimate (± s.e.)** | **F** | **d.f. (1,2)** | ***p*** |
| --- | --- | --- | --- | --- |
| **Linear Model** | | | | |
| *intercept* | 40.461 (0.300) | 58920 | 1, 384.4 | < 0.001 |
| Time Period 2 | 0 (NA) |  |  |  |
| Time Period 3 | 4.733 (0.163) |  |  |  |
| Time Period 4 | 10.99 (0.254) |  |  |  |
| Time Period 5 | 14.143 (0.317) |  |  |  |
| Time Period 6 | 14.496 (0.412) |  |  |  |
| Time Period 7 | 16.348 (0.601) |  |  |  |
| Time Period 8 | 17.405 (0.613) |  |  |  |
| Time Period 9 | 23.077 (0.678) |  |  |  |
| Time Period 10 | 25.805 (0.581) | 429.70 | 8, 604.7 | < 0.001 |
| Protein | 0.207 (0.270) | 0.59 | 1, 525.6 | 0.444 |
| Lipid | 1.090 (0.260) | 17.52 | 1, 457.3 | < 0.001 |
| **Non-Linear Model** | | | | |
| Protein^2^ | -0.527 (1.055) | 0.25 | 1, 956.1 | 0.617 |
| Lipid^2^ | -4.589 (1.218) | 14.19 | 1, 728.0 | < 0.001 |
| Protein*Lipid | 1.085 (0.451) | 5.78 | 1, 701.0 | 0.016 |
| **Interaction Model** | | | | |
| *intercept* | 39.115 (0.402) | 41100.00 | 1, 541.7 | < 0.001 |
| Time Period 2 | 0 (NA) |  |  |  |
| Time Period 3 | 5.301 (0.216) |  |  |  |
| Time Period 4 | 11.941 (0.316) |  |  |  |
| Time Period 5 | 15.107 (0.363) |  |  |  |
| Time Period 6 | 15.231 (0.447) |  |  |  |
| Time Period 7 | 17.094 (0.647) |  |  |  |
| Time Period 8 | 18.242 (0.642) |  |  |  |
| Time Period 9 | 23.681 (0.707) |  |  |  |
| Time Period 10 | 27.306 (0.744) | 431.70 | 8, 601.8 | < 0.001 |
| Protein | 0.152 (0.276) | 1.421 | 1, 520.5 | 0.234 |
| Lipid | 3.546 (0.872) | 20.20 | 1, 757.4 | < 0.001 |
| Lipid^2^ | -4.841 (0.982) | 24.27 | 1, 862.4 | < 0.001 |
| Protein*Lipid | 0.677 (0.265) | 6.51 | 1, 992.6 | 0.011 |
| Time Period 2*Lipid | 0 (NA) |  |  |  |
| Time Period 3*Lipid | 0.866 (0.274) |  |  |  |
| Time Period 4*Lipid | 1.53 (0.381) |  |  |  |
| Time Period 5*Lipid | 1.927 (0.445) |  |  |  |
| Time Period 6*Lipid | 2.254 (0.53) |  |  |  |
| Time Period 7*Lipid | 2.323 (0.678) |  |  |  |
| Time Period 8*Lipid | 2.014 (0.675) |  |  |  |
| Time Period 9*Lipid | 2.608 (0.721) |  |  |  |
| Time Period 10*Lipid | 3.081 (1.022) | 3.15 | 8, 609.2 | 0.002 |

Linear model contained all linear terms, Non-linear model contains all linear terms and the listed non-linear and interaction effects and the Interaction model contains main effects that were significant in previous models (linear and non-linear) and their interactions. Estimates (± s.e.) come from ASreml models, conditional F and *p* obtained through a Wald test.

**Table S16. Outputs from model of the effect of macronutrient intake on female weight.**

|  | **Estimate (± s.e.)** | **F** | **d.f. (1,2)** | ***p*** |
| --- | --- | --- | --- | --- |
| **Linear Model** | | | | |
| *intercept* | 0.796 (0.020) | 2728 | 1, 545.9 | < 0.001 |
| Time Period 2 | 0 (NA) |  |  |  |
| Time Period 3 | 0.334 (0.010) |  |  |  |
| Time Period 4 | 1.073 (0.027) |  |  |  |
| Time Period 5 | 1.329 (0.032) |  |  |  |
| Time Period 6 | 1.417 (0.041) |  |  |  |
| Time Period 7 | 1.590 (0.043) |  |  |  |
| Time Period 8 | 1.920 (0.053) |  |  |  |
| Time Period 9 | 2.630 (0.074) |  |  |  |
| Time Period 10 | 3.187 (0.087) | 319.20 | 8, 564.4 | < 0.001 |
| Protein | 0.039 (0.023) | 2.84 | 1, 756.5 | 0.093 |
| Lipid | 0.031 (0.023) | 1.78 | 1, 567.1 | 0.183 |
| **Quadratic Model** | | | | |
| Protein^2^ | -0.091 (0.093) | 0.75 | 8, 647.2 | 0.651 |
| Lipid^2^ | -0.263 (0.123) | 2.43 | 8, 637.2 | 0.014 |
| Protein*Lipid | 0.127 (0.045) | 1.18 | 8, 642.8 | 0.306 |
| **Interaction Model** | | | | |
| *intercept* | 0.682 (0.045) | 1242 | 1, 606.6 | < 0.001 |
| Time Period 2 | 0 (NA) |  |  |  |
| Time Period 3 | 0.351 (0.024) |  |  |  |
| Time Period 4 | 1.085 (0.036) |  |  |  |
| Time Period 5 | 1.31 (0.038) |  |  |  |
| Time Period 6 | 1.383 (0.048) |  |  |  |
| Time Period 7 | 1.545 (0.052) |  |  |  |
| Time Period 8 | 1.86 (0.061) |  |  |  |
| Time Period 9 | 2.539 (0.082) |  |  |  |
| Time Period 10 | 3.213 (0.255) | 350.90 | 8, 552.8 | < 0.001 |
| Protein | 0.251 (0.081) | 0.38 | 1, 690.2 | 0.539 |
| Lipid | 0.343 (0.11) | 6.89 | 1, 763.6 | 0.009 |
| Protein^2^ | -0.242 (0.096) | 6.33 | 1, 798.1 | 0.012 |
| Lipid^2^ | -0.381 (0.163) | 21.03 | 1, 814.5 | < 0.001 |
| Protein*Lipid | 0.196 (0.048) | 16.44 | 1, 672.9 | < 0.001 |
| Time Period 2*Lipid | 0 (NA) |  |  |  |
| Time Period 3*Lipid | 0.151 (0.057) |  |  |  |
| Time Period 4*Lipid | 0.212 (0.085) |  |  |  |
| Time Period 5*Lipid | 0.399 (0.086) |  |  |  |
| Time Period 6*Lipid | 0.289 (0.118) |  |  |  |
| Time Period 7*Lipid | 0.408 (0.111) |  |  |  |
| Time Period 8*Lipid | 0.497 (0.139) |  |  |  |
| Time Period 9*Lipid | 0.639 (0.195) |  |  |  |
| Time Period 10*Lipid | 1.318 (0.534) | 3.66 | 8, 597.7 | < 0.001 |
| Time Period 2*Lipid^2^ | 0 (NA) |  |  |  |
| Time Period 3*Lipid^2^ | -0.151 (0.106) |  |  |  |
| Time Period 4*Lipid^2^ | -0.156 (0.125) |  |  |  |
| Time Period 5*Lipid^2^ | -0.329 (0.126) |  |  |  |
| Time Period 6*Lipid^2^ | -0.243 (0.147) |  |  |  |
| Time Period 7*Lipid^2^ | -0.354 (0.139) |  |  |  |
| Time Period 8*Lipid^2^ | -0.405 (0.156) |  |  |  |
| Time Period 9*Lipid^2^ | -0.467 (0.194) |  |  |  |
| Time Period 10*Lipid^2^ | -1.365 (1.058) | 1.59 | 8, 633.2 | 0.126 |

Linear model contained all linear terms, Non-linear model contains all linear terms and the listed non-linear and interaction effects and the Interaction model contains main effects that were significant in previous models (linear and non-linear) and their interactions. Estimates (± s.e.) come from ASreml models, conditional F and *p* obtained through a Wald test.

**Table S17. Outputs from model of the sex differences in the effect macronutrient intake on length.**

|  | **Estimate (± s.e.)** | **F** | **d.f. (1,2)** | ***p*** |
| --- | --- | --- | --- | --- |
| *intercept* | 38.81 (0.464) | 48830.00 | 1, 1132.9 | < 0.001 |
| Time Period 2 | 0 (NA) |  |  |  |
| Time Period 3 | 5.605 (0.267) |  |  |  |
| Time Period 4 | 12.023 (0.352) |  |  |  |
| Time Period 5 | 15.368 (0.390) |  |  |  |
| Time Period 6 | 15.723 (0.449) |  |  |  |
| Time Period 7 | 17.564 (0.541) |  |  |  |
| Time Period 8 | 18.696 (0.549) |  |  |  |
| Time Period 9 | 24.164 (0.611) |  |  |  |
| Time Period 10 | 27.494 (0.681) | 728.00 | 8, 1474.5 | < 0.001 |
| Protein | 0.781 (0.682) | 0.06 | 1, 2351.9 | 0.806 |
| Lipid | 3.143 (0.649) | 63.79 | 1, 1500.3 | < 0.001 |
| Protein^2^ | -1.492 (0.820) | 3.309 | 1, 1598.3 | 0.069 |
| Lipid^2^ | -4.030 (0.705) | 32.66 | 1, 1554.7 | < 0.001 |
| Sex (male) | -0.988 (0.358) | 229.90 | 1, 752.9 | < 0.001 |
| Protein*Lipid | 0.628 (0.279) | 5.08 | 1, 1493.4 | 0.024 |
| Time Period 2*Protein | 0 (NA) |  |  |  |
| Time Period 3*Protein | 0.388 (0.332) |  |  |  |
| Time Period 4*Protein | -0.132 (0.430) |  |  |  |
| Time Period 5*Protein | 0.253 (0.480) |  |  |  |
| Time Period 6*Protein | 0.390 (0.531) |  |  |  |
| Time Period 7*Protein | 0.452 (0.581) |  |  |  |
| Time Period 8*Protein | 0.470 (0.587) |  |  |  |
| Time Period 9*Protein | 0.465 (0.609) |  |  |  |
| Time Period 10*Protein | 0.589 (0.754) | 35.65 | 8, 1343.7 | < 0.001 |
| Time Period 2*Lipid | 0 (NA) |  |  |  |
| Time Period 3*Lipid | 1.009 (0.243) |  |  |  |
| Time Period 4*Lipid | 1.653 (0.334) |  |  |  |
| Time Period 5*Lipid | 1.952 (0.374) |  |  |  |
| Time Period 6*Lipid | 2.160 (0.417) |  |  |  |
| Time Period 7*Lipid | 2.150 (0.463) |  |  |  |
| Time Period 8*Lipid | 1.904 (0.471) |  |  |  |
| Time Period 9*Lipid | 2.366 (0.504) |  |  |  |
| Time Period 10*Lipid | 2.587 (0.668) | 1.52 | 8, 1492.9 | 0.144 |
| Time Period 2*Sex (m) | 0 (NA) |  |  |  |
| Time Period 3*Sex (m) | -0.411 (0.233) |  |  |  |
| Time Period 4*Sex (m) | -2.345 (0.311) |  |  |  |
| Time Period 5*Sex (m) | -4.556 (0.347) |  |  |  |
| Time Period 6*Sex (m) | -5.417 (0.442) |  |  |  |
| Time Period 7*Sex (m) | -5.504 (0.567) |  |  |  |
| Time Period 8*Sex (m) | -5.065 (0.578) |  |  |  |
| Time Period 9*Sex (m) | -6.353 (0.669) |  |  |  |
| Time Period 10*Sex (m) | -8.072 (0.648) | 4.50 | 8, 1448.4 | < 0.001 |

Estimates (± s.e.) come from ASreml models, conditional F and *p* obtained through a Wald test. For sex comparisons females are the reference level.

**Table S18. Outputs from model of the sex differences in the effect macronutrient intake on weight.**

|  | **Estimate (± s.e.)** | **F** | **d.f. (1,2)** | ***p*** |
| --- | --- | --- | --- | --- |
| *intercept* | 0.692 (0.031) | 2778 | 1, 1475.3 | < 0.001 |
| Time Period 2 | 0 (NA) |  |  |  |
| Time Period 3 | 0.333 (0.017) |  |  |  |
| Time Period 4 | 1.088 (0.027) |  |  |  |
| Time Period 5 | 1.335 (0.029) |  |  |  |
| Time Period 6 | 1.414 (0.038) |  |  |  |
| Time Period 7 | 1.584 (0.039) |  |  |  |
| Time Period 8 | 1.905 (0.046) |  |  |  |
| Time Period 9 | 2.594 (0.06) |  |  |  |
| Time Period 10 | 3.084 (0.116) | 757.4 | 8, 1340.9 | < 0.001 |
| Protein | 0.222 (0.053) | 0.323 | 1, 1511 | 0.570 |
| Lipid | 0.213 (0.071) | 19.55 | 1, 1498.7 | < 0.001 |
| Protein^2^ | -0.287 (0.063) | 20.39 | 1, 2208.4 | < 0.001 |
| Lipid^2^ | -0.169 (0.105) | 35.16 | 1, 2112 | < 0.001 |
| Protein*Lipid | 0.134 (0.028) | 22.04 | 1, 1775.1 | < 0.001 |
| Sex (Male) | -0.028 (0.02) | 0.10 | 1, 778.3 | < 0.001 |
| Time Period 2*Lipid | 0 (NA) |  |  |  |
| Time Period 3*Lipid | 0.216 (0.035) |  |  |  |
| Time Period 4*Lipid | 0.317 (0.051) |  |  |  |
| Time Period 5*Lipid | 0.454 (0.056) |  |  |  |
| Time Period 6*Lipid | 0.445 (0.079) |  |  |  |
| Time Period 7*Lipid | 0.498 (0.074) |  |  |  |
| Time Period 8*Lipid | 0.556 (0.088) |  |  |  |
| Time Period 9*Lipid | 0.687 (0.112) |  |  |  |
| Time Period 10*Lipid | 1.569 (0.255) | 11.31 | 8, 1455.4 | < 0.001 |
| Time Period 2*Lipid^2^ | 0 (NA) |  |  |  |
| Time Period 3*Lipid^2^ | -0.257 (0.066) |  |  |  |
| Time Period 4*Lipid^2^ | -0.3 (0.078) |  |  |  |
| Time Period 5*Lipid^2^ | -0.377 (0.083) |  |  |  |
| Time Period 6*Lipid^2^ | -0.373 (0.098) |  |  |  |
| Time Period 7*Lipid^2^ | -0.403 (0.094) |  |  |  |
| Time Period 8*Lipid^2^ | -0.436 (0.103) |  |  |  |
| Time Period 9*Lipid^2^ | -0.503 (0.118) |  |  |  |
| Time Period 10*Lipid^2^ | -1.939 (0.467) | 4.13 | 8, 1521.8 | < 0.001 |
| Time Period 2*Sex (m) | 0 (NA) |  |  |  |
| Time Period 3*Sex (m) | -0.031 (0.015) |  |  |  |
| Time Period 4*Sex (m) | -0.27 (0.031) |  |  |  |
| Time Period 5*Sex (m) | -0.413 (0.033) |  |  |  |
| Time Period 6*Sex (m) | -0.378 (0.043) |  |  |  |
| Time Period 7*Sex (m) | -0.442 (0.042) |  |  |  |
| Time Period 8*Sex (m) | -0.582 (0.052) |  |  |  |
| Time Period 9*Sex (m) | -0.86 (0.071) |  |  |  |
| Time Period 10*Sex (m) | -1.169 (0.087) | 37.86 | 8, 1235.8 | < 0.001 |

Estimates (± s.e.) come from ASreml models, conditional F and *p* obtained through a Wald test. For sex comparisons females are the reference level.

**Table S19.** **Outputs from model of the effect of macronutrient intake on male condition index.**

|  | **Estimate (± s.e.)** | **F** | **d.f. (1,2)** | ***p*** |
| --- | --- | --- | --- | --- |
| **Linear Model** | | | | |
| *intercept* | 0.022 (0.009) | 10.09 | 1, 459.1 | < 0.001 |
| Time Period 2 | 0 (NA) |  |  |  |
| Time Period 3 | 0.003 (0.008) |  |  |  |
| Time Period 4 | -0.012 (0.011) |  |  |  |
| Time Period 5 | 0.002 (0.012) |  |  |  |
| Time Period 6 | 0.033 (0.017) |  |  |  |
| Time Period 7 | -0.079 (0.016) |  |  |  |
| Time Period 8 | -0.031 (0.014) |  |  |  |
| Time Period 9 | -0.048 (0.015) |  |  |  |
| Time Period 10 | 0.010 (0.013) | 11.56 | 8, 678.7 | < 0.001 |
| Protein | -0.003 (0.006) | 0.19 | 1, 462.8 | 0.667 |
| Lipid | 0.041 (0.006) | 38.61 | 1, 439.9 | < 0.001 |
| **Non-Linear Model** | | | | |
| Protein^2^ | -0.038 (0.035) | 1.15 | 1, 533.0 | 0.285 |
| Lipid^2^ | -0.056 (0.030) | 3.52 | 1, 421.9 | 0.061 |
| Protein*Lipid | 0.021 (0.014) | 2.18 | 1, 389.7 | 0.140 |
| **Interaction Model** | | | | |
| *intercept* | 0.107 (0.073) | < 0.00 | 1, 449.0 | 0.959 |
| Time Period 2 | 0 (NA) |  |  |  |
| Time Period 3 | -0.110 (0.067) |  |  |  |
| Time Period 4 | -0.094 (0.073) |  |  |  |
| Time Period 5 | -0.085 (0.073) |  |  |  |
| Time Period 6 | -0.050 (0.074) |  |  |  |
| Time Period 7 | -0.209 (0.074) |  |  |  |
| Time Period 8 | -0.136 (0.074) |  |  |  |
| Time Period 9 | -0.145 (0.074) |  |  |  |
| Time Period 10 | -0.294 (0.095) | 12.43 | 8, 668.8 | < 0.001 |
| Protein | -0.046 (0.102) | 0.08 | 1, 559.3 | 0.774 |
| Lipid | 0.070 (0.056) | 30.62 | 1, 517.9 | < 0.001 |
| Protein^2^ | 0.254 (0.256) | 0.01 | 1, 569.0 | 0.922 |
| Lipid^2^ | -0.098 (0.102) | 10.87 | 1, 544.3 | 0.001 |
| Time Period 2*Protein | 0 (NA) |  |  |  |
| Time Period 3*Protein | 0.090 (0.096) |  |  |  |
| Time Period 4*Protein | 0.023 (0.103) |  |  |  |
| Time Period 5*Protein | 0.048 (0.104) |  |  |  |
| Time Period 6*Protein | -0.017 (0.115) |  |  |  |
| Time Period 7*Protein | 0.130 (0.111) |  |  |  |
| Time Period 8*Protein | 0.078 (0.108) |  |  |  |
| Time Period 9*Protein | 0.076 (0.110) |  |  |  |
| Time Period 10*Protein | 0.310 (0.151) | 1.81 | 8, 633.9 | 0.072 |
| Time Period 2*Lipid | 0 (NA) |  |  |  |
| Time Period 3*Lipid | 0.036 (0.051) |  |  |  |
| Time Period 4*Lipid | -0.001 (0.058) |  |  |  |
| Time Period 5*Lipid | 0.035 (0.060) |  |  |  |
| Time Period 6*Lipid | 0.127 (0.077) |  |  |  |
| Time Period 7*Lipid | 0.263 (0.074) |  |  |  |
| Time Period 8*Lipid | 0.134 (0.067) |  |  |  |
| Time Period 9*Lipid | 0.064 (0.072) |  |  |  |
| Time Period 10*Lipid | 0.298 (0.100) | 4.51 | 8, 623.6 | < 0.001 |
| Time Period 2*Protein^2^ | 0 (NA) |  |  |  |
| Time Period 3*Protein^2^ | -0.334 (0.231) |  |  |  |
| Time Period 4*Protein^2^ | -0.218 (0.251) |  |  |  |
| Time Period 5*Protein^2^ | -0.258 (0.255) |  |  |  |
| Time Period 6*Protein^2^ | -0.209 (0.259) |  |  |  |
| Time Period 7*Protein^2^ | -0.328 (0.258) |  |  |  |
| Time Period 8*Protein^2^ | -0.282 (0.257) |  |  |  |
| Time Period 9*Protein^2^ | -0.286 (0.258) |  |  |  |
| Time Period 10*Protein^2^ | -0.774 (0.330) | 2.00 | 8, 641.7 | 0.045 |
| Time Period 2*Lipid^2^ | 0 (NA) |  |  |  |
| Time Period 3*Lipid^2^ | -0.012 (0.091) |  |  |  |
| Time Period 4*Lipid^2^ | 0.051 (0.100) |  |  |  |
| Time Period 5*Lipid^2^ | 0.036 (0.102) |  |  |  |
| Time Period 6*Lipid^2^ | -0.048 (0.112) |  |  |  |
| Time Period 7*Lipid^2^ | -0.131 (0.111) |  |  |  |
| Time Period 8*Lipid^2^ | -0.04 (0.107) |  |  |  |
| Time Period 9*Lipid^2^ | 0.019 (0.110) |  |  |  |
| Time Period 10*Lipid^2^ | -0.437 (0.182) | 3.65 | 8, 624.7 | < 0.001 |

Linear model contained all linear terms, Non-linear model contains all linear terms and the listed non-linear and interaction effects and the Interaction model contains main effects and any significant and their interactions. Estimates (± s.e.) come from ASreml models, conditional F and *p* obtained through a Wald test.

**Table S20. Outputs from models of the effect of macronutrient intake on female condition index.**

|  | **Estimate (± s.e.)** | **F** | **d.f. (1,2)** | ***p*** |
| --- | --- | --- | --- | --- |
| **Linear Model** | | | | |
| *intercept* | 0.028 (0.009) | 13.30 | 1, 499.2 | < 0.001 |
| Time Period 2 | 0 (NA) |  |  |  |
| Time Period 3 | -0.022 (0.008) |  |  |  |
| Time Period 4 | -0.043 (0.011) |  |  |  |
| Time Period 5 | -0.072 (0.011) |  |  |  |
| Time Period 6 | -0.098 (0.016) |  |  |  |
| Time Period 7 | 0.001 (0.022) |  |  |  |
| Time Period 8 | -0.059 (0.023) |  |  |  |
| Time Period 9 | -0.041 (0.021) |  |  |  |
| Time Period 10 | -0.036 (0.017) | 7.83 | 8, 496.9 | < 0.001 |
| Protein | 0.022 (0.007) | 9.65 | 1, 510.2 | 0.002 |
| Lipid | 0.017 (0.006) | 8.26 | 1, 488.8 | 0.004 |
| **Non-Linear Model** | | | | |
| Protein^2^ | -0.06 (0.034) | 3.04 | 1, 711.6 | 0.082 |
| Lipid^2^ | -0.018 (0.034) | 0.29 | 1, 527.6 | 0.592 |
| Protein*Lipid | 0.012 (0.012) | 0.89 | 1, 521.1 | 0.346 |

Estimates (± s.e.) come from ASreml models, conditional F and *p* obtained through a Wald test. Linear model contained all linear terms, Non-linear model contains all linear terms and the listed non-linear and interaction effects. There were no significant interaction effects for female condition.

**Table S21. Outputs from model of the sex differences in the effect macronutrient intake on condition index.**

|  | **Estimate (± s.e.)** | **F** | **d.f. (1,2)** | ***p*** |
| --- | --- | --- | --- | --- |
| *intercept* | 0.013 (0.010) | 2.43 | 1, 1018.6 | 0.119 |
| Time Period 2 | 0 (NA) |  |  |  |
| Time Period 3 | -0.017 (0.009) |  |  |  |
| Time Period 4 | -0.030 (0.012) |  |  |  |
| Time Period 5 | -0.058 (0.012) |  |  |  |
| Time Period 6 | -0.086 (0.017) |  |  |  |
| Time Period 7 | 0.001 (0.021) |  |  |  |
| Time Period 8 | -0.050 (0.020) |  |  |  |
| Time Period 9 | -0.031 (0.020) |  |  |  |
| Time Period 10 | -0.018 (0.019) | 3.62 | 8, 1177.6 | < 0.001 |
| Protein | 0.020 (0.007) | 2.51 | 1, 979.4 | 0.113 |
| Lipid | 0.028 (0.012) | 21.47 | 1, 1607.6 | < 0.001 |
| Lipid^2^ | -0.040 (0.013) | 8.49 | 1, 1151.4 | 0.004 |
| Sex (male) | -0.005 (0.013) | 25.12 | 1, 1029.2 | < 0.001 |
| Time Period 2*Lipid | 0 (NA) |  |  |  |
| Time Period 3*Lipid | 0.007 (0.009) |  |  |  |
| Time Period 4*Lipid | 0.020 (0.011) |  |  |  |
| Time Period 5*Lipid | 0.030 (0.012) |  |  |  |
| Time Period 6*Lipid | 0.020 (0.015) |  |  |  |
| Time Period 7*Lipid | 0.065 (0.016) |  |  |  |
| Time Period 8*Lipid | 0.043 (0.015) |  |  |  |
| Time Period 9*Lipid | 0.030 (0.015) |  |  |  |
| Time Period 10*Lipid | 0.043 (0.019) | 3.08 | 8, 1184.8 | 0.002 |
| Sex (m)*Lipid | 0.025 (0.009) | 4.55 | 1, 992.9 | 0.033 |
| Sex (m)*Protein | -0.021 (0.010) | 7.91 | 1, 939.1 | 0.005 |
| Time Period 2*Sex (m) | 0 (NA) |  |  |  |
| Time Period 3*Sex (m) | 0.029 (0.012) |  |  |  |
| Time Period 4*Sex (m) | 0.032 (0.016) |  |  |  |
| Time Period 5*Sex (m) | 0.074 (0.017) |  |  |  |
| Time Period 6*Sex (m) | 0.130 (0.024) |  |  |  |
| Time Period 7*Sex (m) | -0.085 (0.027) |  |  |  |
| Time Period 8*Sex (m) | 0.023 (0.026) |  |  |  |
| Time Period 9*Sex (m) | -0.007 (0.025) |  |  |  |
| Time Period 10*Sex (m) | 0.048 (0.021) | 13.28 | 8, 1174.6 | < 0.001 |

Estimates (± s.e.) come from ASreml models, conditional F and *p* obtained through a Wald test. For sex comparisons females are the reference level.

**Table S22. Outputs from linear regressions of diet on food intake (gday^-1^).**

|  | **DF** | **F** | ***p*** |
| --- | --- | --- | --- |
| **Males** | | | |
| Diet | 4, 13 | 88.93 | < 0.001 |
| Size | 1, 13 | 38.39 | < 0.001 |
| Diet: Size | 4, 9 | 3.16 | 0.07 |
| **Females** | | | |
| Diet | 4, 14 | 97.38 | < 0.001 |
| Size | 1, 14 | 97.22 | < 0.001 |
| Diet: Size | 4, 10 | 2.97 | 0.074 |
| **Sex-Specific** | | | |
| Sex | 1, 28 | 0.31 | 0.585 |

**Table S23. Posthoc tukey comparison for the effect of diet (protein : lipid) on intake (gday^-1^).**

|  | **Males** | | **Females** | |
| --- | --- | --- | --- | --- |
| **Diet (P:L)** | **Estimate (s.e.)** | ***p*** | **Estimate (s.e.)** | ***p*** |
| 4.6:1 - 10.2:1 | 0.113 (0.006) | < 0.001 | 0.046 (0.006) | < 0.001 |
| 2.5:1 - 10.2:1 | 0.071 (0.006) | < 0.001 | 0.065 (0.006) | < 0.001 |
| 8.5:1 - 10.2:1 | 0.093 (0.006) | < 0.001 | 0.113 (0.006) | < 0.001 |
| 1.6:1 - 10.2:1 | 0.047 (0.007) | < 0.001 | 0.030 (0.006) | 0.002 |
| 2.5:1 - 4.6:1 | -0.042 (0.006) | < 0.001 | 0.019 (0.006) | 0.050 |
| 8.5:1 - 4.6:1 | -0.020 (0.006) | 0.056 | 0.066 (0.006) | < 0.001 |
| 1.6:1 - 4.6:1 | -0.065 (0.007) | < 0.001 | -0.016 (0.006) | 0.104 |
| 8.5:1 - 2.5:1 | 0.022 (0.006) | 0.034 | 0.048 (0.006) | < 0.001 |
| 1.6:1 - 2.5:1 | -0.023 (0.007) | 0.037 | -0.035 (0.006) | < 0.001 |
| 1.6:1 - 8.5:1 | -0.045 (0.007) | < 0.001 | -0.083 (0.006) | < 0.001 |

**Table S24. Number of individuals in each diet treatment.**

| **Diet** | **Sex** | **Size** | **100%** | **75%** | **50%** | **Total** |
| --- | --- | --- | --- | --- | --- | --- |
| 1 | F | L | 9 | 10 | 10 | 29 |
|  |  | S | 10 | 9 | 10 | 29 |
|  | M | L | 11 | 10 | 10 | 31 |
|  |  | S | 10 | 10 | 10 | 30 |
| 2 | F | L | 10 | 10 | 10 | 30 |
|  |  | S | 10 | 10 | 10 | 30 |
|  | M | L | 10 | 10 | 9 | 29 |
|  |  | S | 10 | 9 | 10 | 29 |
| 3 | F | L | 12 | 10 | 11 | 33 |
|  |  | S | 10 | 10 | 10 | 30 |
|  | M | L | 8 | 10 | 9 | 27 |
|  |  | S | 9 | 10 | 10 | 29 |
| 4 | F | L | 11 | 10 | 10 | 31 |
|  |  | S | 9 | 9 | 9 | 27 |
|  | M | L | 9 | 10 | 10 | 29 |
|  |  | S | 11 | 10 | 11 | 32 |
| 5 | F | L | 9 | 10 | 9 | 28 |
|  |  | S | 10 | 10 | 10 | 30 |
|  | M | L | 11 | 10 | 10 | 31 |
|  |  | S | 10 | 10 | 10 | 30 |
|  | | **Total** | 199 | 197 | 198 | **594** |

Initially 10 individuals of each sex and size class (L = Large, S = Small) were assigned to each treatment. However, due to mortality immediately prior to the experiment and some errors in molecular sexing (N=10), the final sample sizes were as below.

**Table S25. Table showing the content (%) of different ingredients in the five diets used in this experiment.**

|  | **Diet (P:L)** | | | | |
| --- | --- | --- | --- | --- | --- |
| **Ingredient** | **10.2 : 1** | **8.5 : 1** | **4.6 : 1** | **2.5 : 1** | **1.6 : 1** |
| Herring Meal | 90.24 | 41.65 | 79.83 | 69.42 | 41.65 |
| Corn Starch (Filler) | 6.26 | 54.85 | 11.17 | 13.61 | 38.77 |
| Lecithin | 1.00 | 1.00 | 1.00 | 1.00 | 1.00 |
| Vitamin /mineral premix | 1.00 | 1.00 | 1.00 | 1.00 | 1.00 |
| ASTX (10% carophyll pink) | 1.00 | 1.00 | 1.00 | 1.00 | 1.00 |
| CMC binder | 0.50 | 0.50 | 0.50 | 0.50 | 0.50 |
| Fish Oil | 0.00 | 0.00 | 5.50 | 13.47 | 16.08 |
| Energy | 13.8 | 7.1 | 14.8 | 16.3 | 12.4 |

Diets are described by their protein : lipid ratio (P:L). Herring meal is both a source of protein and lipid, therefore fish oil was only required in diets with high lipid contents. Energy represents the usable energy in the diet. Macronutrient values are percentages of raw materials (g) in the diet.

**Table S26. Table showing the number of individuals alive at each weighing time period, for each sex.**

|  | **Diet (P:L)** | | | | | | | | | |
| --- | --- | --- | --- | --- | --- | --- | --- | --- | --- | --- |
|  | **10.2:1** | | **8.5:1** | | **4.6:1** | | **2.5:1** | | **1.6:1** | |
| **Time Period** | **M** | **F** | **M** | **F** | **M** | **F** | **M** | **F** | **M** | **F** |
| (1) Nov 2014 | 61 | 59 | 60 | 59 | 60 | 60 | 57 | 63 | 61 | 59 |
| (2) Dec 2014 | 54 | 56 | 56 | 58 | 53 | 59 | 56 | 60 | 60 | 55 |
| (3) Jan 2015 | 43 | 55 | 52 | 57 | 51 | 57 | 54 | 58 | 54 | 51 |
| (4) Mar 2015 | 38 | 52 | 51 | 52 | 47 | 55 | 51 | 57 | 53 | 47 |
| (5) June 2015 | 35 | 46 | 48 | 47 | 47 | 53 | 50 | 50 | 51 | 47 |
| (6) Aug 2015* | 13 | 17 | 22 | 20 | 20 | 25 | 14 | 23 | 27 | 25 |
| (7) Oct 2015 | 18 | 22 | 35 | 30 | 31 | 27 | 40 | 25 | 41 | 27 |
| (8) Dec 2015 | 16 | 19 | 32 | 26 | 31 | 26 | 39 | 20 | 39 | 22 |
| (9) Feb 2016 | 16 | 16 | 31 | 20 | 27 | 24 | 38 | 18 | 37 | 19 |
| (10) May 2016 | 14 | 13 | 27 | 15 | 26 | 20 | 36 | 15 | 35 | 16 |
| Jul 2016** | 11 | 6 | 18 | 11 | 20 | 9 | 27 | 7 | 27 | 8 |
| Sep 2016** | 9 | 4 | 11 | 7 | 17 | 3 | 14 | 4 | 15 | 2 |
| Nov 2016** | 8 | 2 | 8 | 1 | 13 | 1 | 10 | 3 | 11 | 1 |
| Dec 2016** | 8 | 2 | 8 | 1 | 10 | 1 | 9 | 2 | 11 | 1 |

*Some data was lost for the August 2015 weighing.

**Data excluded from final analysis due to low sample size

**Table S27.** **Outputs from LME models exploring the linear and non-linear effects of age, protein and lipid on male nuptial colour (red intensity).**

|  | **Estimate (± s.e.)** | **χ^2^** | ***p*** |
| --- | --- | --- | --- |
| **Linear Model** | | | |
| *intercept* | 0.477 (0.005) |  |  |
| Age | 0.256 (0.009) |  | < 0.001 |
| Age^2^ | -0.258 (0.009) | 593.06 | < 0.001 |
| Age Last | 0.003 (0.002) | 3.24 | 0.071 |
| Protein | 0.002 (0.002) | 0.61 | 0.435 |
| Lipid | 0.005 (0.003) | 3.95 | 0.047 |
| **Non-Linear Model** | | | |
| Age Last^2^ | 0.014 (0.023) | 0.37 | 0.544 |
| Protein^2^ | -0.024 (0.015) | 2.57 | 0.109 |
| Lipid^2^ | -0.038 (0.013) | 8.40 | 0.004 |
| Protein*Lipid | 0.012 (0.005) | 5.49 | 0.019 |
| **Interaction Model** | | | |
| *intercept* | 0.474 (0.005) |  |  |
| Age | 0.257 (0.009) |  |  |
| Age^2^ | -0.259 (0.009) |  |  |
| Age Last | 0.003 (0.002) |  |  |
| Protein | < 0.000 (0.003) |  |  |
| Lipid | 0.029 (0.010) |  |  |
| Lipid^2^ | -0.025 (0.010) |  |  |
| Protein*Lipid | 0.004 (0.003) |  |  |
| Age*Lipid | < -0.00 (0.002) | 0.00 | 0.989 |
| Age*Protein | 0.002 (0.001) | 2.69 | 0.101 |

All models included the non-linear effect of Age and the *p* value for Age was estimated using the package lmerTest as it is only significant when Age^2^ was included in the model. Linear model contained all linear terms and Age^2^, Non-linear model contains all linear terms and the listed non-linear and interaction effects and the Interaction model contains main effects that were significant in previous models (linear and non-linear) and their interactions.

Supplementary Figures


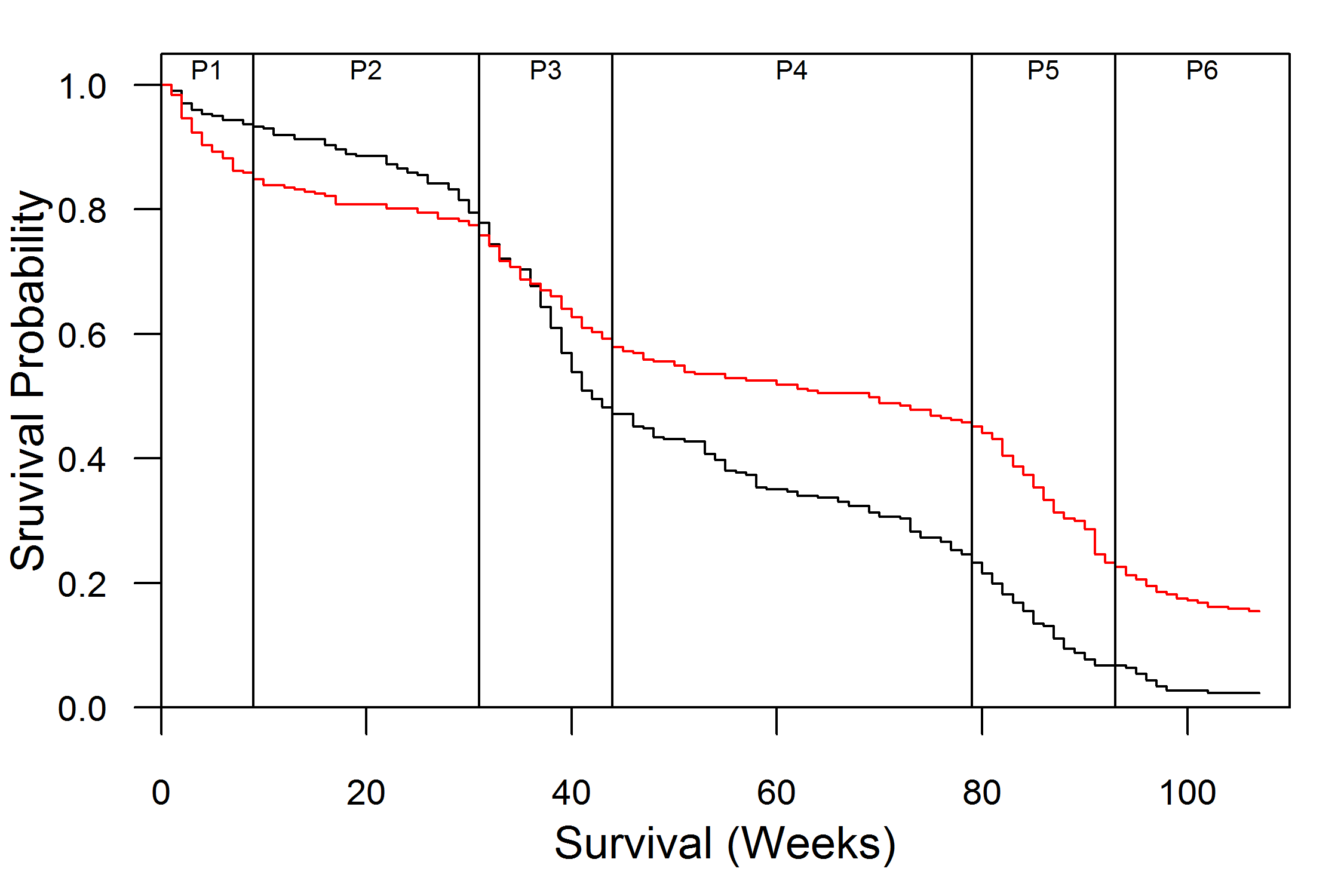


**Fig S1.** Kaplan-Meier Survival plot showing the relationship between time (weeks) and proportion of individuals alive. The 6 time periods that were included in the survival models are represented as P1 – P6. The black line represents females and the red line males.

**
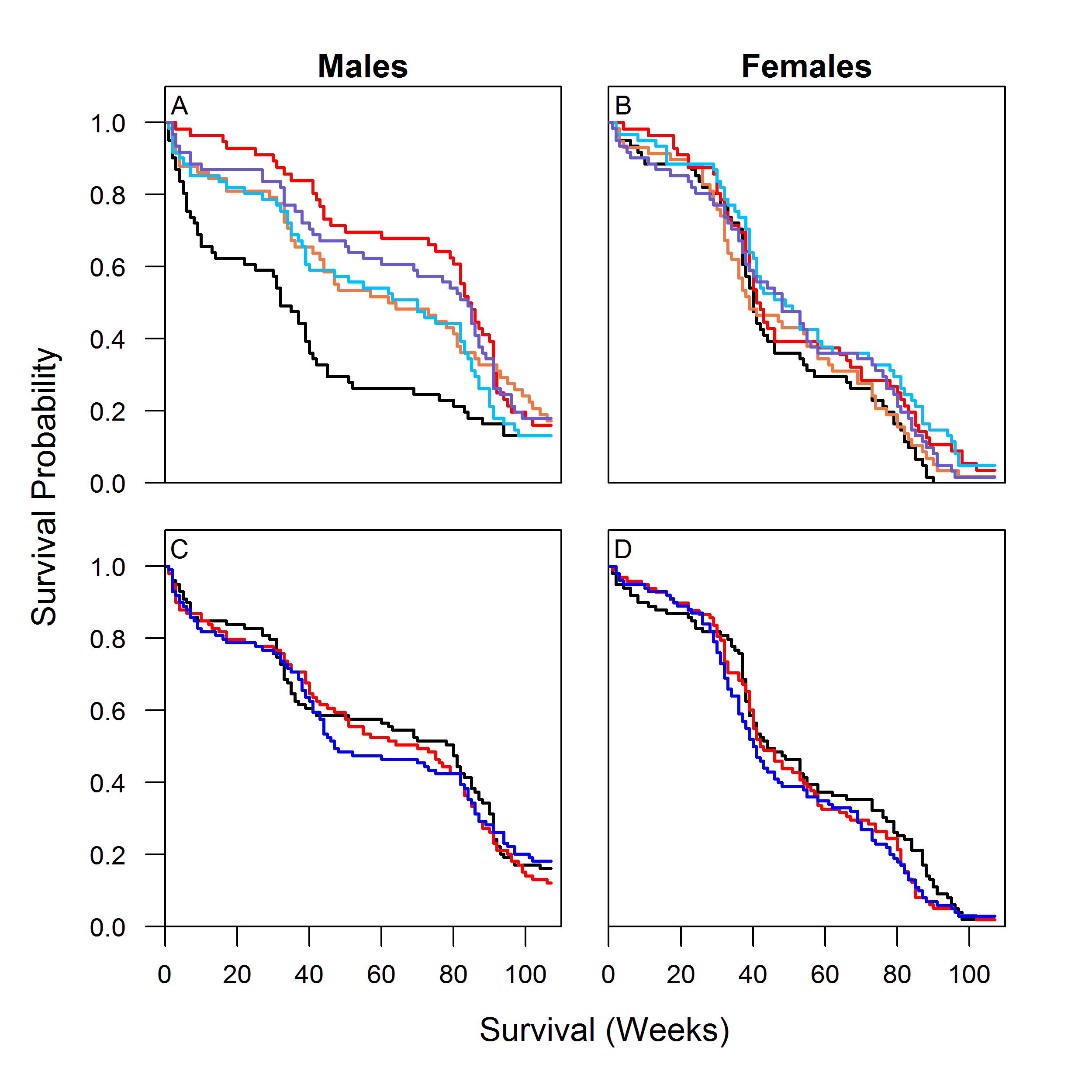
**

**Fig S2**. Kaplan-Meier survival curves for the effect of Diet (A and B) and Food Level (C and D) on survival in weeks. For panels A and B, the five diets are as follows: 10.2:1 – Black, 8.5:1 – Blue, 4.6:1 – Orange, 2.5:1 – Red and 1.6:1 – Purple. For panels C and D, the 3 food levels are: 100% (fully fed) – Blue, 75% – Red and 50% – Black. There was a significant effect of diet in both sexes (all *p* < 0.043) but no effect of level (*p* > 0.5). There were also significant differences between the sexes (*p* < 0.001) with males (A and C) living longer than females (B and D).


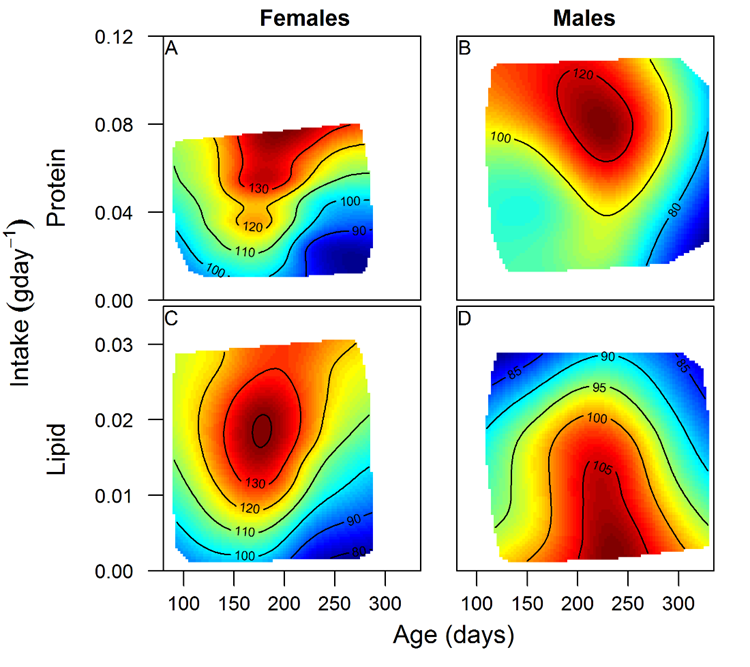


**Fig S3** Non-parametric thin-plate spline contour visualisations showing the effects of protein and lipid intake on reproductive senescence. Panel response surfaces are as follows: (A) and (C) female egg production (number of eggs produced at each clutch), (B) and (D) male courtship (time spent courting (s)). All plots have age on the x axis with (A and B) having protein (gday^-1^) and (C and D) having lipid (gday^-1^) on the y axis. (A) is the same plot as Fig 3B, however we present it again here to allow for comparison with other figures.


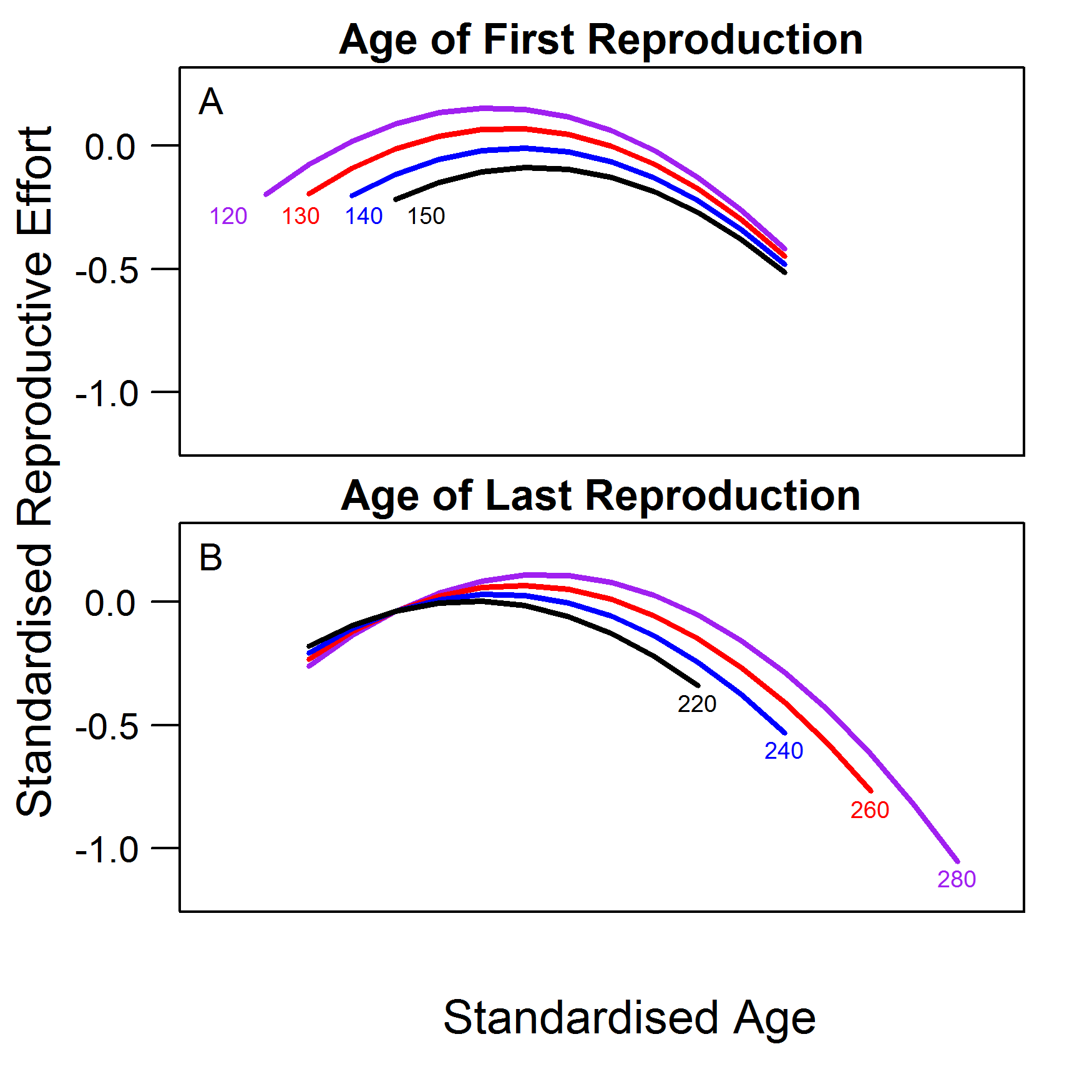


**Fig S4.** Predicted age-specific trajectories of female reproductive senescence. Curves represent the predicted reproductive effort for groups of individuals with different (A) age of first and (B) age of last reproductive events (ages indicated by numbers next to the curves). There was a significant effect of both age of first and age of last reproductive event on patterns of female reproductive senescence. The ages selected for each plot were chosen to cover the 90^th^ percentile of the data. For panel (A) the senescence lines end at the mean age of last reproductive event, for panel (B) the curves start at the mean age of first reproductive event. Age and reproductive effort are standardised values (mean of 0 and standard deviation of 1).


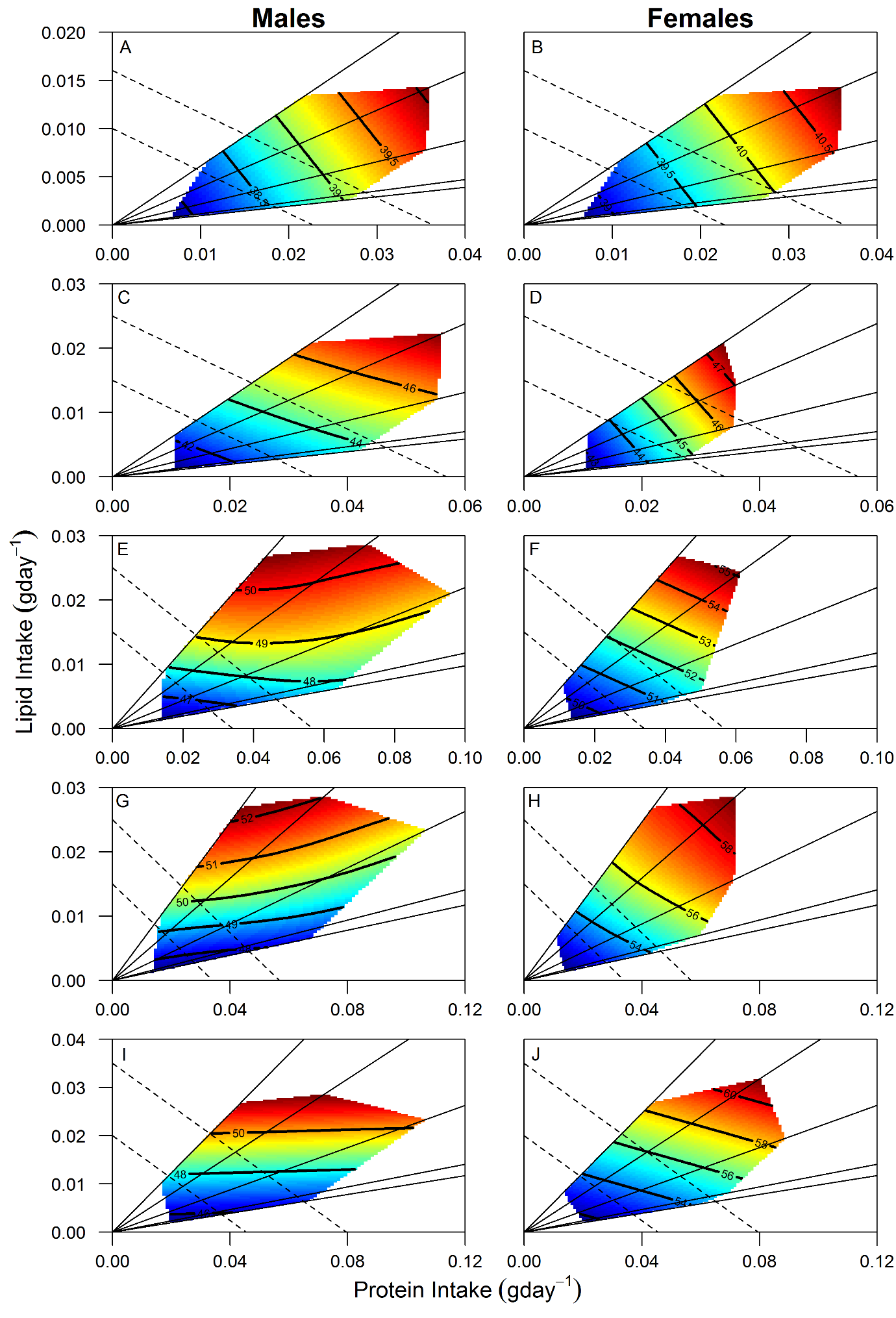


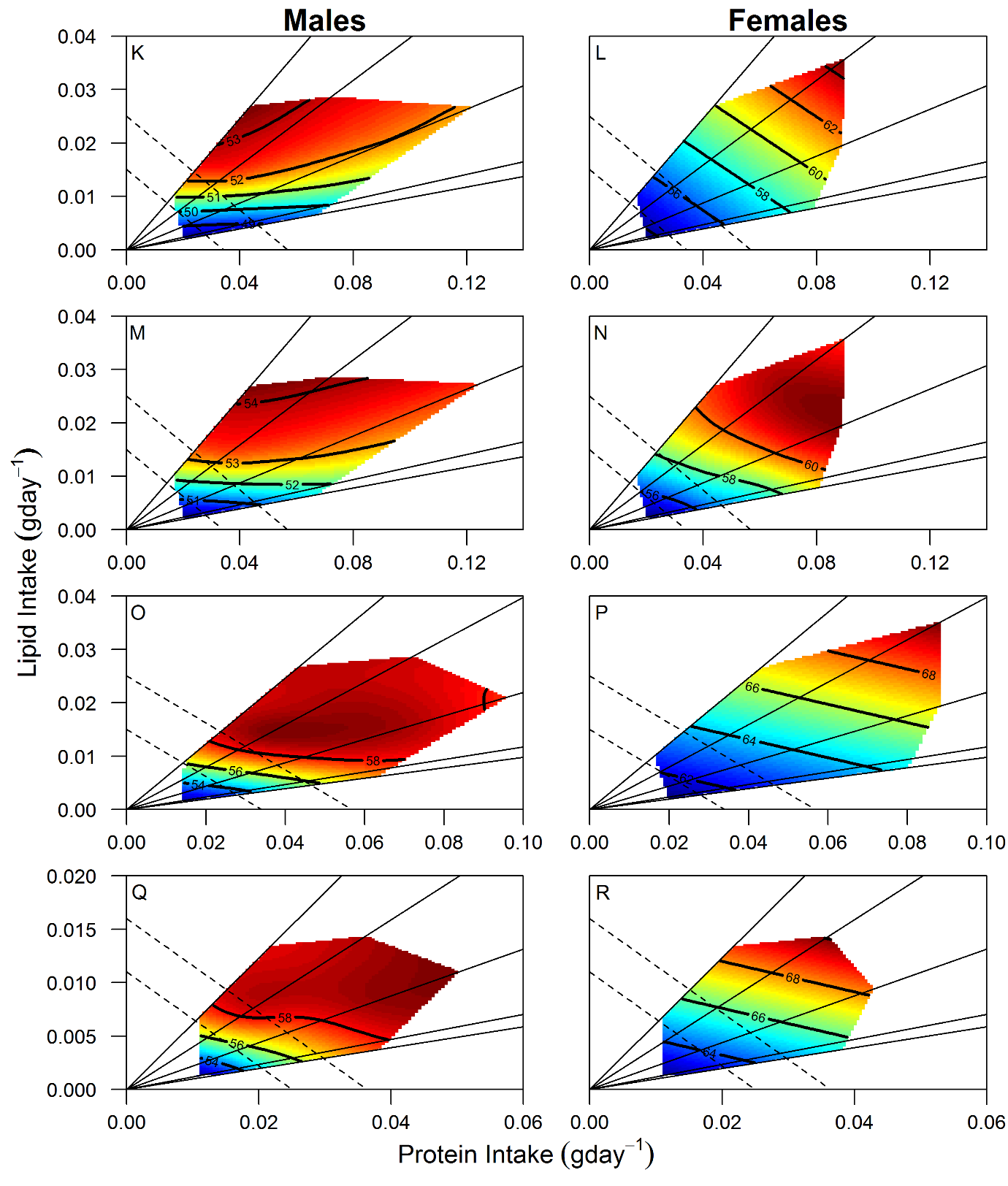


**Fig S5.** Non-parametric thin-plate spline contour visualisations for the effect of protein and lipid intake (gday^-1^) on length at different time points. Time point (see Table S26 for timings): 2 (A-B), 3 (C-D), 4 (E-F), 5 (G-H), 6 (I-J), 7 (K-L), 8 (M-N), 9 (O-P) and 10 (Q-R). Time period 1 is not presented as this was the initial length of fish, prior to dietary treatment. See main texts for discussion of results and Fig. 4.

**
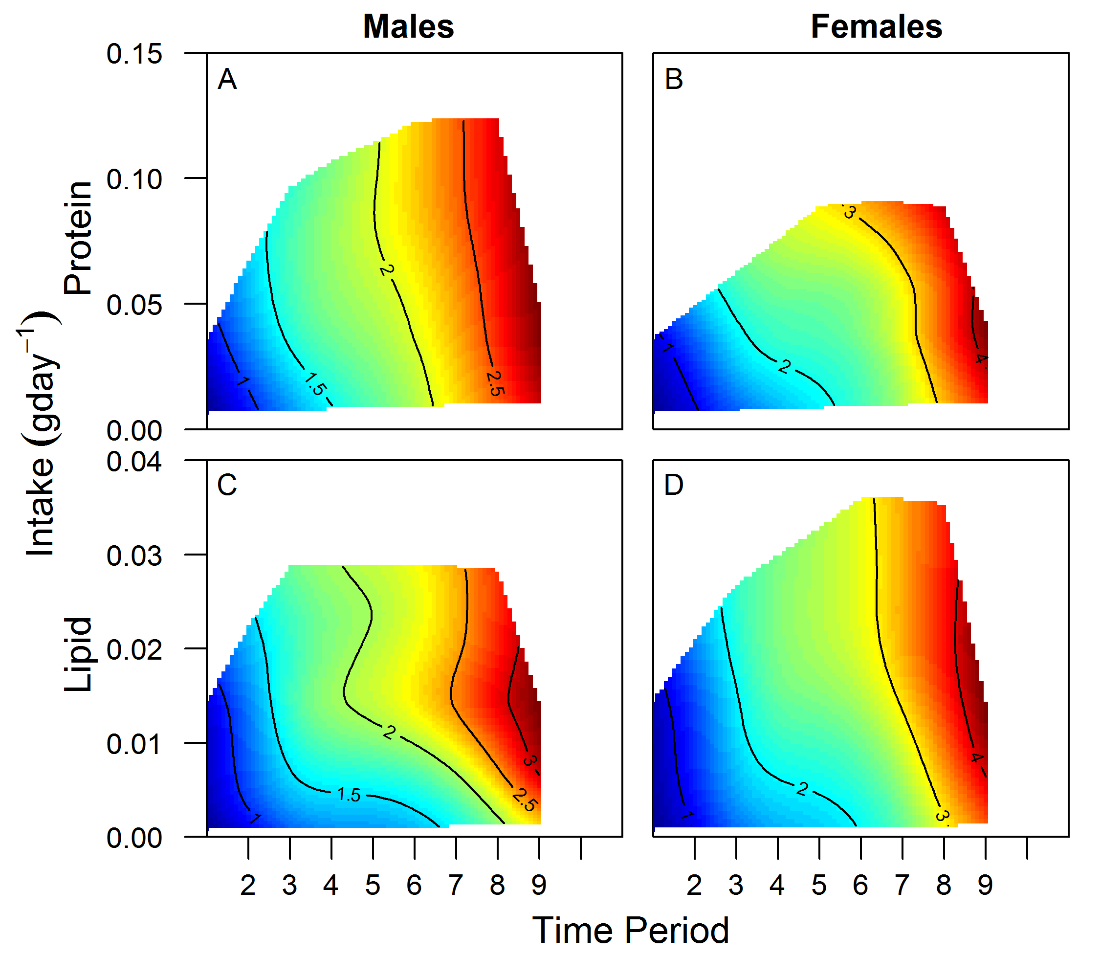
**

**Fig S6.** Non-parametric thin-plate spline contour visualisations for the effect of protein and lipid intake (gday^-1^) on weight (g) across batches. Panel response surfaces as follows: (A) effect of protein on male weight, (B) effect of protein on female weight, (C) effect of lipid on male weight, and (D) effect of lipid on female weight. There were no differences in the effect of macronutrients between the sexes.


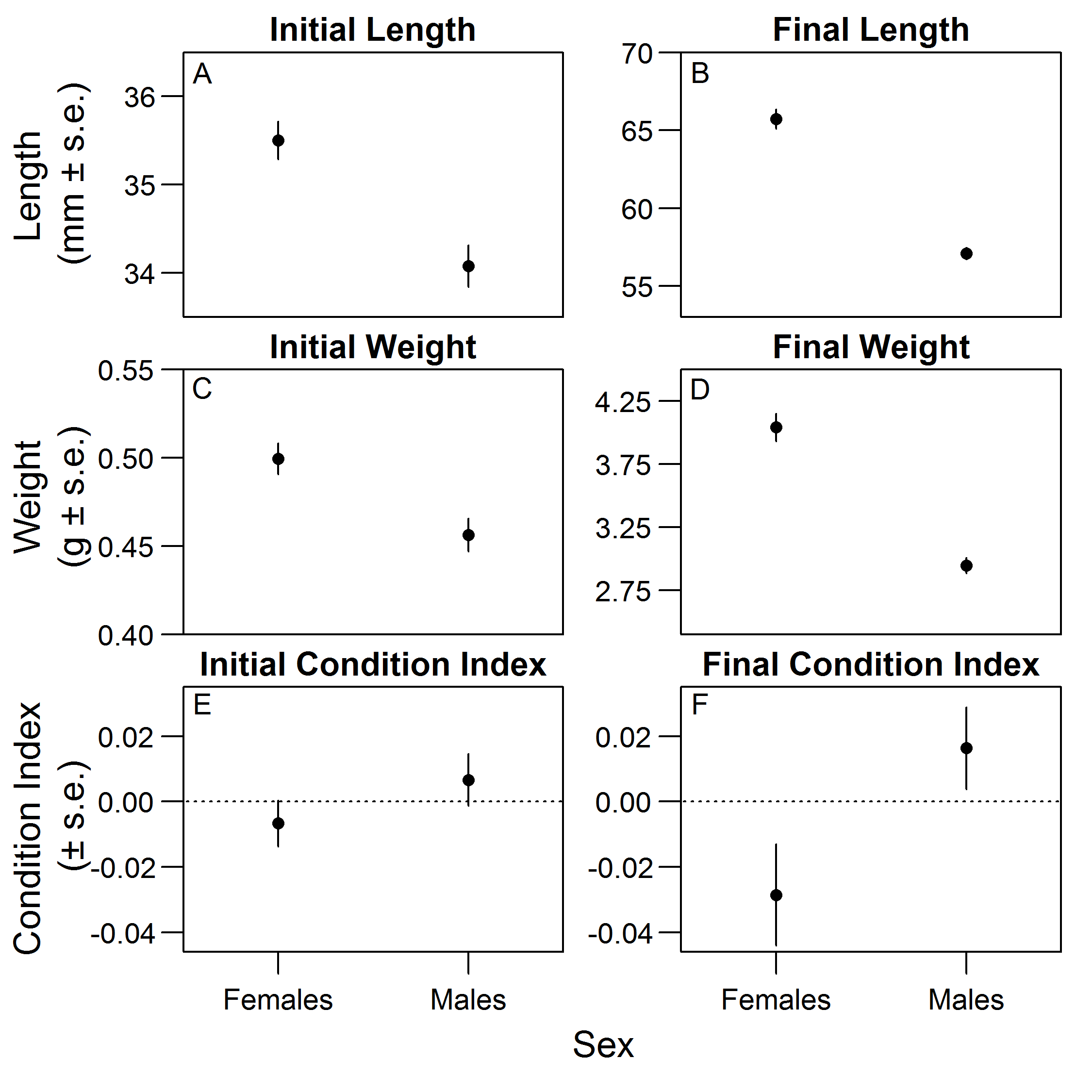


**Fig S7.** Sex differences in growth and condition. Values represent the mean (± s.e.) for each sex. Panels as follows: (A) initial length, (B) final length, (C) initial weight, (D) final weight, (E) initial condition index and (F) final condition index. For condition index, zero is average condition (dashed line), with a positive value indicating a better than average condition, a negative value worse than average. There were significant differences in initial length and weight and these differences increased through the course of the experiment. There was no difference in initial condition but at final measurement females were in significantly worse condition than males.


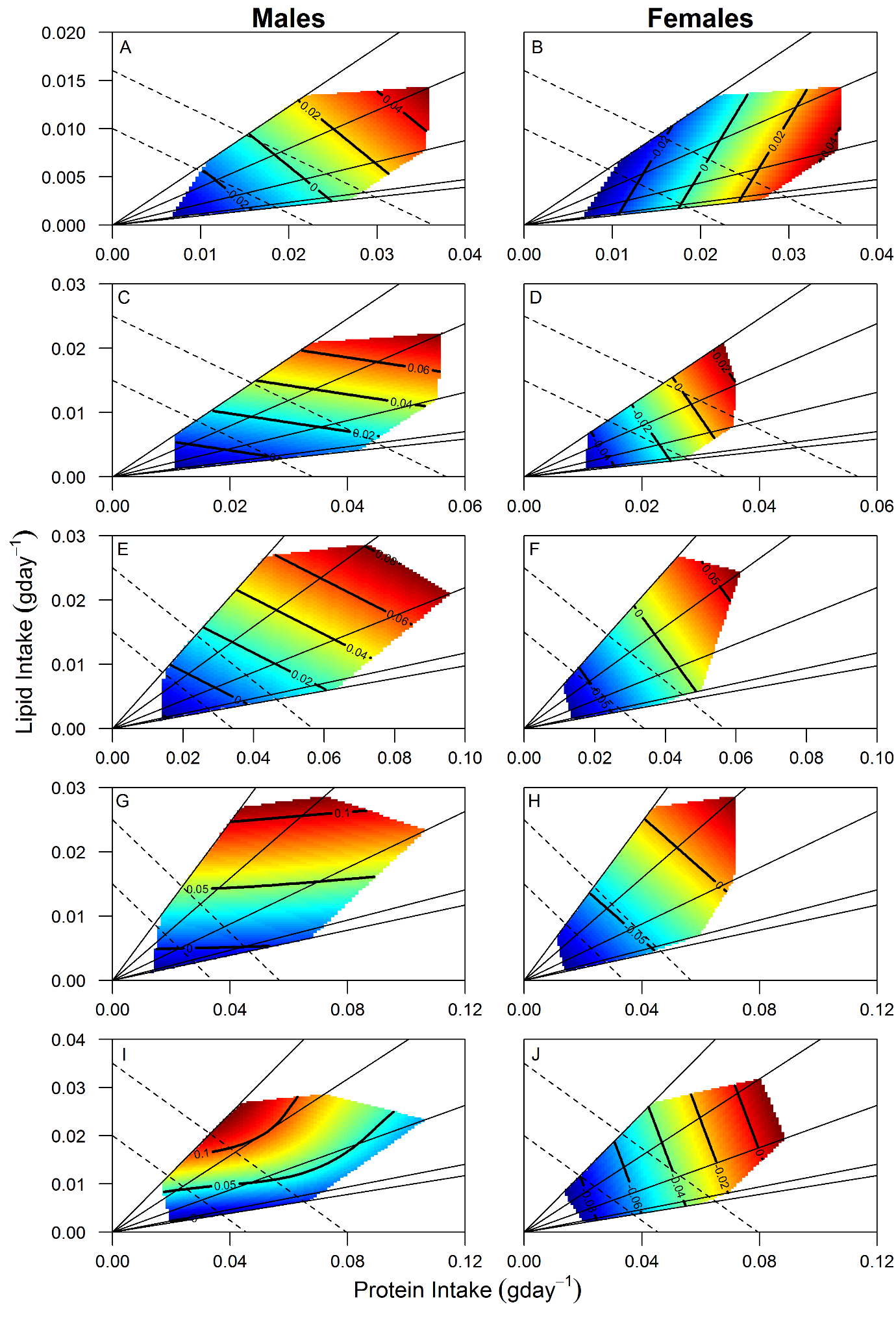


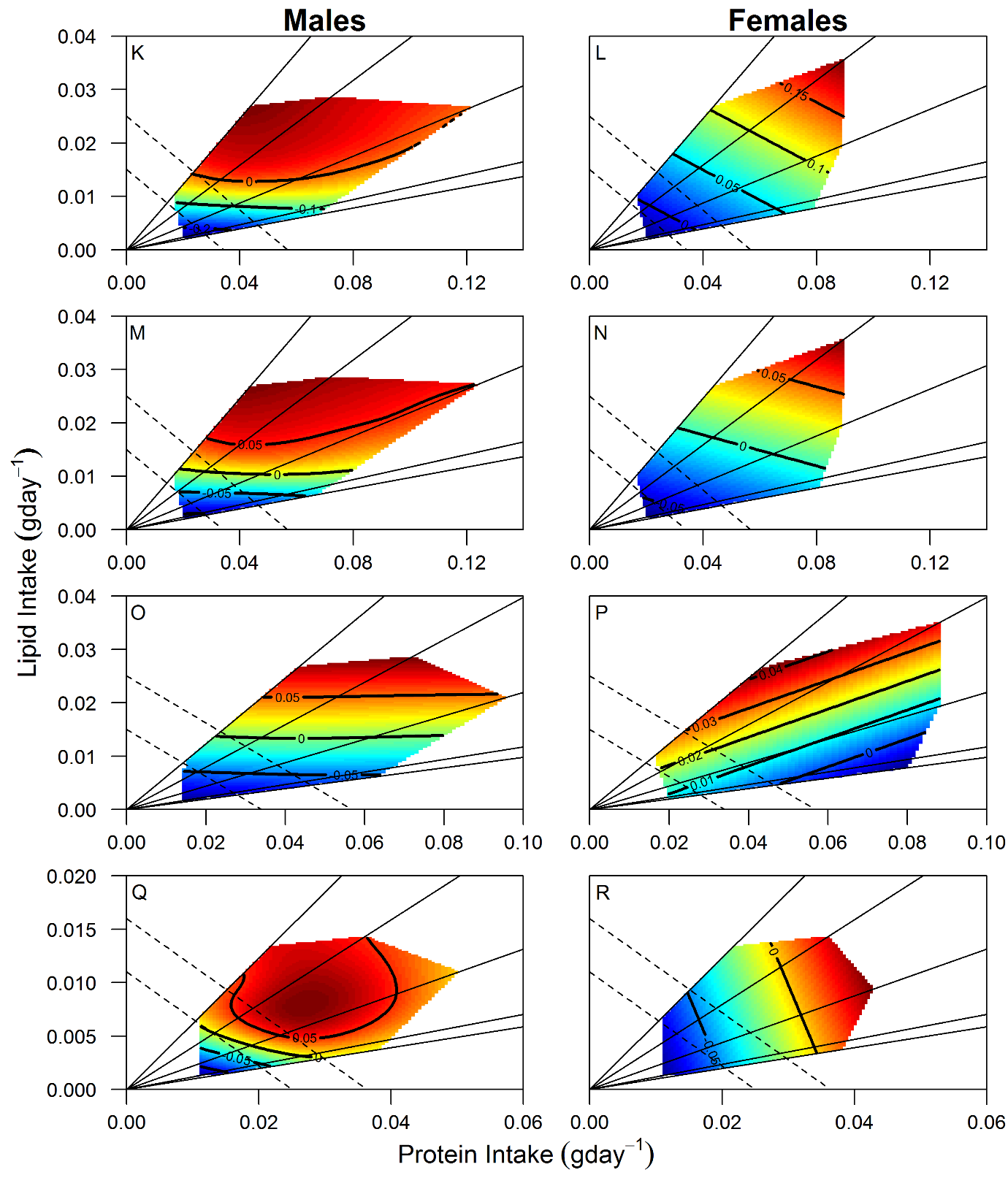


**Fig S8.** Non-parametric thin-plate spline contour visualisations for the effect of protein and lipid intake (gday^-1^) on body condition at different time points. Time point (see Table S26 for timings): 2 (A-B), 3 (C-D), 4 (E-F), 5 (G-H), 6 (I-J), 7 (K-L), 8 (M-N), 9 (O-P) and 10 (Q-R). Time period 1 is not presented as this was the initial condition of fish, prior to dietary treatment. See main texts for discussion of results and Fig. 5.


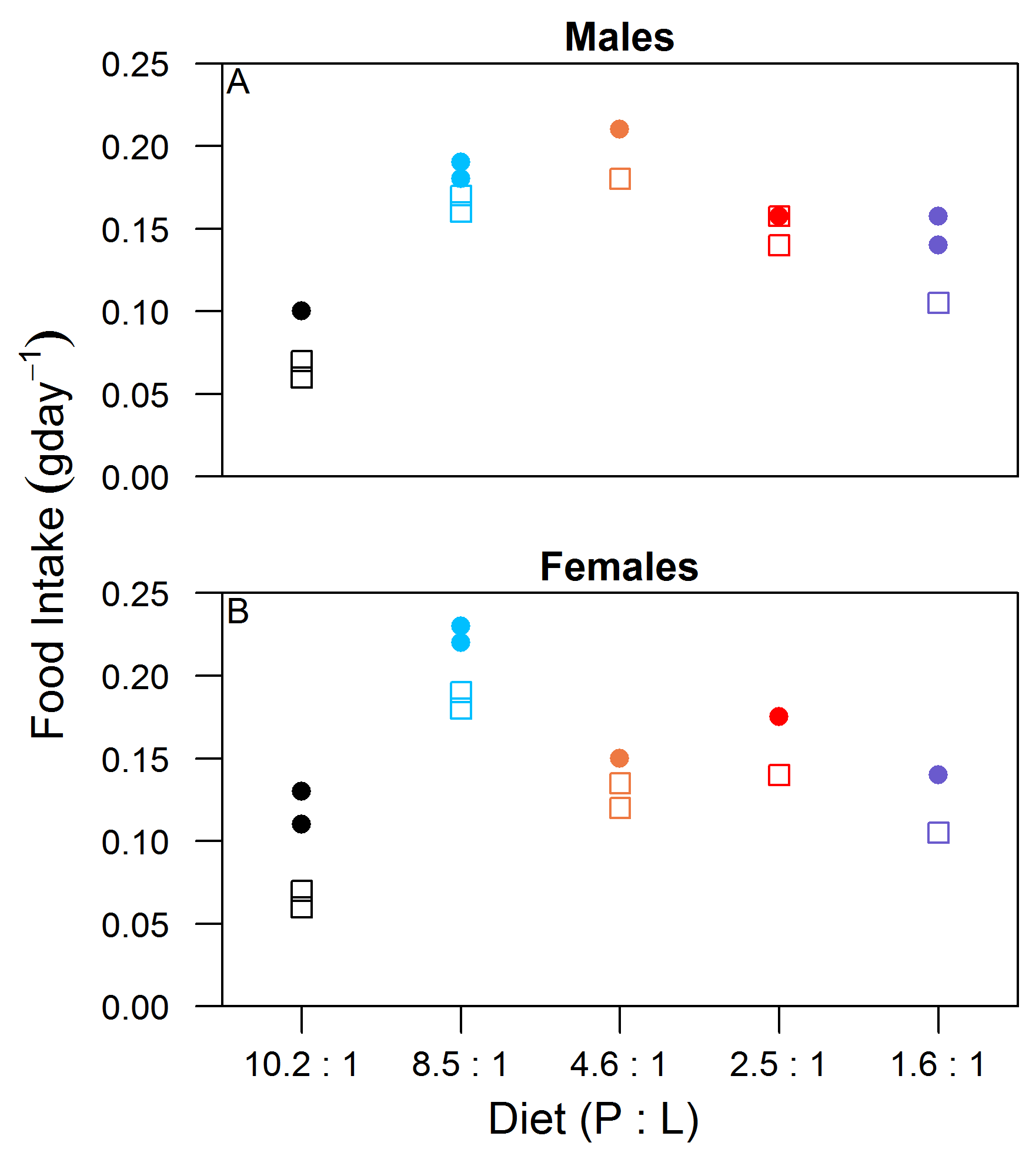


**Fig S9.** Intakes (gday^-1^) for (A) male and (B) female sentinel fish on different diets (ratio of protein : lipid) during the stable period of intakes (195 days), where intake rates were at their highest. Closed circles represent large individuals and open squares represent small individuals. Colours represent the five dietary ratios of protein : lipid There was no difference in intake rates between the sexes. However, there were significant effects of both diet and size, with fish on diets with a balanced ratio of protein : lipid consuming more food, and large fish eating more than small fish (see Tables S19 and S20).


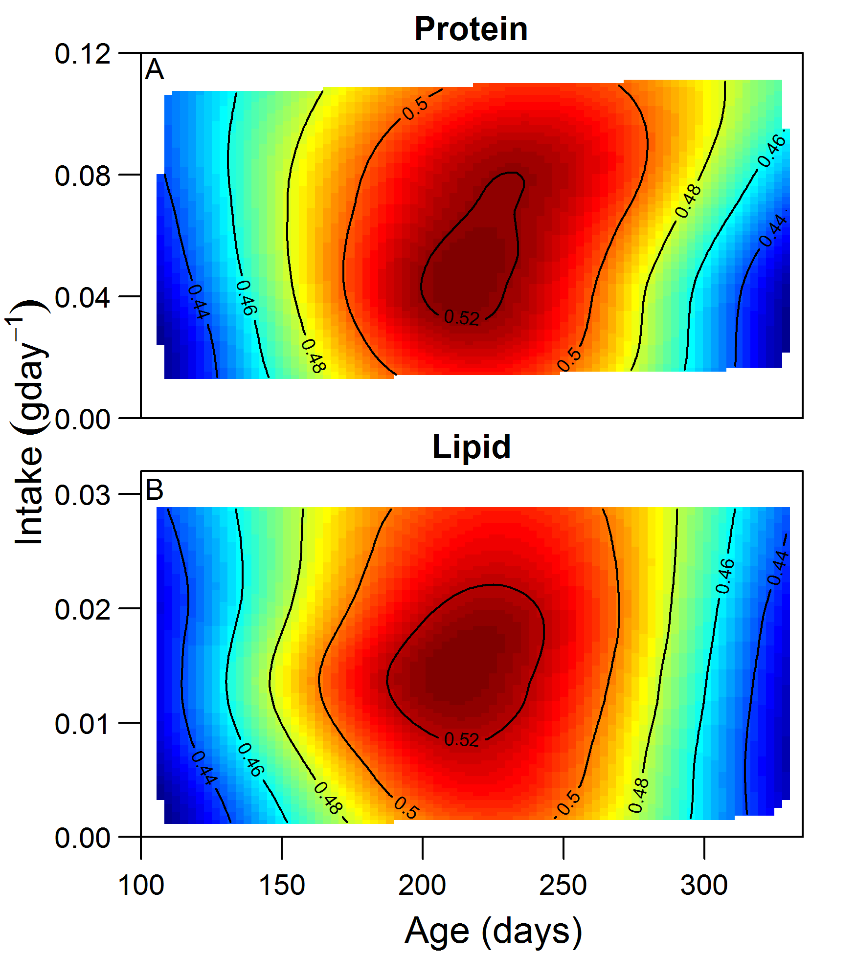


**Fig S10.** Non-parametric thin-plate spline contour plots showing the effect of age (days) and macronutrient intake on male breeding colour (red intensity) across the first breeding season. Panel (A) protein intake (gday^-1^), panel (B) lipid intake (gday^-1^).
